# Beyond TLS Presence: A Functional Framework Integrating Maturity, Location, and Immune Context

**DOI:** 10.3390/cancers18152393

**Published:** 2026-07-25

**Authors:** Jakub Kleinrok, Kamil Rusztyn, Marta Druszcz, Weronika Pająk, Filip Gajewski, Miłosz Badach, Agnieszka Korolczuk, Maciej Mazur

**Affiliations:** 1 Chair and Department of Clinical Pathomorphology, Medical University of Lublin, Jaczewskiego 8b, 20-090 Lublin, Poland; marta.druszcz@interia.pl (M.D.); 62108@umlub.edu.pl (W.P.); 63380@umlub.edu.pl (F.G.); mimbad@interia.pl (M.B.);; 2 Faculty of Chemistry, University of Warsaw, Ludwika Pasteura 1, 02-093 Warszawa, Poland; mmazur@chem.uw.edu.pl; 3 Faculty of Medicine, Medical University of Warsaw, Ul. Zwirki I Wigury 61, 02-091 Warsaw, Poland

**Keywords:** tertiary lymphoid structures, solid tumours, tumour microenvironment, B cells, germinal centres, immunotherapy, pathology, biomarkers

## Abstract

TLSs are ectopic, non-encapsulated aggregates of immune cells that develop de novo in non-lymphoid tissues in response to persistent antigenic stimulation and have emerged as clinically relevant features of many solid tumours. However, conventional TLS assessment based on presence/absence, density, or simplified maturation scales does not adequately explain why TLSs are associated with favourable, neutral, or even adverse clinical outcomes across tumour types and treatment settings. In this review, we synthesise the current biological, spatial, and clinical evidence and argue that TLSs should be interpreted not as static histologic findings but as functional immune niches shaped by three interacting axes: structural maturity, spatial localisation, and the functional immune context. We discuss how mature germinal centre-positive TLSs often reflect coordinated B-cell–T-cell cooperation and sustained antigen-driven anti-tumour immunity, whereas partially organised or suppressive TLSs may display transitional or immunoregulatory properties. On this basis, we propose a pragmatic, pathology-oriented conceptual framework that groups TLSs into three simplified functional states: TLS-A, representing mature effector TLSs with germinal centre activity; TLS-B, representing organised but incompletely matured or functionally intermediate TLSs; and TLS-C, representing TLSs dominated by regulatory or suppressive immune programs. We further place these states within recurrent tumour microenvironment archetypes and outline the rationale for a “proposed functional TLS score” integrating histopathologic and molecular readouts. Rather than introducing a definitive biological taxonomy, this framework is intended as a translational model for harmonising TLS interpretation, refining biomarker development, and supporting future studies on prognosis, immunotherapy response, and standardised pathology reporting in solid tumours. Because this framework is derived from a narrative synthesis of published evidence rather than from formal validation, it should be regarded as hypothesis-generating and requires prospective, tumour-specific validation before clinical implementation.

## Simple Summary

Tertiary lymphoid structures are immune-cell clusters that can form inside or around tumours. In many cancers, their presence has been linked with better immune activity and an improved response to immunotherapy, but this is not always the case. Some structures may be mature and support anti-tumour immunity, whereas others may remain incomplete or even contribute to immune suppression. In this review, we explain why tertiary lymphoid structures should not be assessed only as present or absent. Instead, their maturity, location, and immune-cell composition should be considered together. We propose a practical framework dividing these structures into three functional states: pro-immunogenic, intermediate, and suppressive. This approach may help researchers and pathologists to interpret tertiary lymphoid structures more consistently and may support future studies on prognosis, immunotherapy response, and standardised tumour assessment.

## 1. Introduction

### 1.1. Definition, Formation, and Architecture of TLS

TLSs are ectopic, non-encapsulated aggregates of immune cells that develop de novo in non-lymphoid tissues in response to persistent antigenic stimulation and chronic inflammation [1,2,3]. These structures frequently arise in perivascular niches and display varying degrees of organisation, ranging from loose lymphocytic aggregates to highly ordered structures with the partial or well-defined segregation of B-cell and T-cell zones. In more mature TLSs, B-cell areas may contain germinal centres (GCs). Adjacent T-cell zones contain dendritic cells (DCs) and stromal components, which support antigen presentation and lymphocyte organisation. Together, these features contribute to local immune coordination [3,4,5]. Endothelial remodelling and ectopic lymphoid chemokine expression, including CCL19, CCL21, and CXCL13, promote the formation of high endothelial venules (HEVs). They facilitate lymphocyte and DC recruitment into TLSs [3,6]. In the microenvironments of solid tumours, TLSs may therefore constitute specialised sites of local activation and coordination of anti-tumour immune responses [3].

### 1.2. TLSs in Solid Tumours

TLSs have been identified across a broad spectrum of solid tumours, including those of the lung, colon, ovary, kidney, stomach, oesophagus, and liver [7,8]. However, their biological and clinical relevance varies across tumour types and appears to depend on maturation, localisation, and cellular composition [9].

An increasing number of observations indicate that mature TLSs, particularly structures resembling secondary follicles and containing GCs, are of particular clinical relevance, as, in selected solid tumours, they have been associated with more favourable outcomes, including prolonged progression-free survival (PFS) and overall survival (OS). In addition, meta-analytic and cohort-based data suggest that TLSs may reflect a more immunologically active tumour microenvironment and may serve as a useful predictive biomarker of the response to immunotherapy, especially immune checkpoint inhibitor (ICI) treatment [10,11]. This is particularly relevant in the context of B-cell immunity, as TLSs may support local antigen presentation, germinal centre reactions, and B–T-cell cooperation, although the extent of this activity may vary across individual structures [12].

### 1.3. From TLS Presence to Functional Interpretation

Previous approaches to TLSs have focused primarily on quantitative parameters and simple maturity scales, which only partially capture the biological complexity of these structures. Moreover, depending on the tumour type and microenvironmental organisation, intratumoural and peritumoural TLSs may be associated with different clinical outcomes, and TLS abundance alone does not always translate into the same biological effect. This indicates that conventional quantitative and spatial parameters are insufficient for a comprehensive interpretation of the significance of TLSs in solid tumours [1]. TLS heterogeneity is also shaped by the cellular composition. Immune and myeloid subsets may exert distinct and sometimes opposing biological effects. Therefore, TLS function depends not only on their presence and architecture but also on the dominant cellular interactions within these structures [12]. An additional, often overlooked dimension is the local cytokine, chemokine, and metabolic context, which may either promote TLS induction and maturation or direct them toward immature, dysfunctional, or suppressive states [5]. Furthermore, TLSs are not static structures but rather dynamic immunological niches whose organisation, cellular composition, and functional activity may be reshaped over time under the influence of tumour microenvironment evolution and treatment [5]. Taken together, these observations suggest that TLSs in solid tumours do not represent a uniform histological category but rather form a functional spectrum with diverse biological and clinical properties, thereby justifying the need for a more integrated classification [1,5].

### 1.4. Aim of the Review

This review aims to synthesise the current biological, spatial, and clinical evidence of TLSs in solid tumours and to reframe TLSs not as a purely binary, density-based, or maturity-based biomarker but as a spectrum of functional states shaped by architectural organisation, the anatomical location, and the immune context. Based on this integrative perspective, we propose a pragmatic, pathology-oriented conceptual framework that groups TLSs into three simplified functional states (TLS-A, TLS-B, and TLS-C), intended not as a definitive biological taxonomy but as a conceptual model for organising existing evidence across heterogeneous tumour settings. We further outline the rationale for a “proposed functional TLS score” that combines histopathologic and molecular features and may serve as a foundation for future translational studies, biomarker refinement, and more standardised TLS reporting. Consistent with the narrative and conceptual nature of this review, the proposed TLS-A/B/C states are intended to organise the existing evidence and guide future validation, rather than to serve as fixed diagnostic categories.

## 2. Methodological Approach

This article is a narrative and conceptual review rather than a systematic review or meta-analysis. The review was developed to synthesise biological, spatial, and clinical evidence relevant to TLS interpretation in solid tumours and to formulate a pathology-oriented reporting framework. A targeted literature search was performed in PubMed/MEDLINE, Scopus, and Google Scholar for articles published between 1 February 2010 and 1 April 2026, with a final literature update on 1 April 2026.

Search terms combined phrases related to “tertiary lymphoid structures”, “TLS”, “solid tumours”, “tumour microenvironment”, “B cells”, “germinal centres”, “TLS maturation”, “intratumoural TLS”, “peritumoural TLS”, “immunotherapy”, “multiplex immunohistochemistry”, “spatial transcriptomics”, “digital pathology”, and “TLS gene signature”. Eligible publications included original studies, translational analyses, clinical cohorts, methodological studies, and recent reviews addressing TLS morphology, maturation, localisation, immune composition, prognostic or predictive relevance, therapeutic modulation, or assessment methods in solid tumours. We excluded studies unrelated to solid tumours, articles without relevant TLS-specific data, non-English publications, conference abstracts with insufficient detail, duplicates, and studies focused exclusively on non-neoplastic inflammatory or autoimmune diseases, unless they provided essential mechanistic context for TLS formation or function.

Because the aim was conceptual synthesis, no protocol was registered, no PRISMA flow diagram was prepared, no formal risk-of-bias assessment was performed, and no quantitative meta-analysis was attempted. Study selection was narrative and relevance-based, with priority given to studies that informed at least one of the three framework axes—structural maturity, spatial localisation, or immune context—or that reported prognostic, predictive, mechanistic, or methodological data relevant to TLS assessment in solid tumours.

Literature screening and selection were performed by three authors, and disagreements regarding relevance or interpretation were resolved by discussion rather than by a formal independent screening procedure.

The evidence was synthesised around three axes: structural maturity, spatial localisation, and functional immune context. On this basis, we proposed a pathology-oriented framework dividing TLSs into TLS-A, TLS-B, and TLS-C, and a modular Functional TLS Scoring Framework integrating histopathology, the TLS burden, localisation, the immune-cell composition, and optional molecular signatures. This framework is intended as a conceptual model for translational studies and standardised reporting, rather than as a validated clinical scoring system.

## 3. Biology of TLSs and B-Cell Immunity in Solid Tumours

TLSs are formations that develop in chronically inflamed tissues, including the tumour microenvironment (TME). They are spatially organised compartments, similar to secondary lymphoid organs (SLOs) but developed in distinct pathological conditions. Their presence suggests a continuous local immune response. The structural and cellular heterogeneity of TLSs leads directly to diverse roles in tumour progression and anti-tumour immunity. Therefore, understanding the mechanisms regulating TLS formation, maturation, and function is critical.

### 3.1. Induction and Development of TLSs

TLS development is an inflammatory reaction to various conditions, such as infections, autoimmune diseases, tissue transplantation, and malignancies. This is one of the key differences observed in TLS formation in contrast to SLOs, where development occurs in a sequential manner during embryogenesis. Moreover, SLOs are characterised by clearly segregated T- and B-cell compartments, whereas TLSs display considerable heterogeneity in their development, ranging from a diffuse infiltrate composed only of T cells to T- and B-cell accumulations without distinct compartmentalisation and to mature and well-organised structures containing germinal centres (GCs) [1,13,14].

TLSs are composed predominantly of T cells (CD3+), B cells (CD20+), fibroblasts, follicular dendritic cells (FDCs, CD21+), macrophages, and high endothelial venules (HEVs) expressing peripheral node addressin (PNAd). The main factor initiating TLS formation is chronic inflammation, which leads to leukocyte infiltration. The subsequent activation of these cells, together with the activation of stromal cells, causes the production of a wide variety of pro-inflammatory and pro-lymphangiogenic cytokines. In addition, stromal cells undergo phenotypic polarisation in the TME [1,13,14,15].

Cytokine-recruited lymphoid tissue inducer (LTi) cells trigger TLS formation through interaction with lymphotoxin beta receptor (LTβR) and tumour necrosis factor receptor 1 (TNFR1) expressed on mesenchymal stromal cells. LTi cells subsequently give signals to acquire the lymphoid tissue organiser (LTo) phenotype by stromal cells, mainly through lymphotoxin α1β2 (LTα1β2) and tumour necrosis factor (TNF) signalling. This interaction induces LTo cells to produce adhesion molecules such as vascular endothelial growth factor C (VEGF-C), vascular cell adhesion molecule 1 (VCAM-1), and intercellular adhesion molecule 1 (ICAM-1), as well as chemokines, including IL-7, C-C chemokine ligand type 12 (CCL12), CCL19, CCL21, and C-X-C chemokine ligand type 13 (CXCL13). These molecules are important for HEV formation, lymphocyte recruitment, and B- and T-cell compartmentalisation in TLSs. In addition, it has been reported that a minimum density of 70 B cells/mm2 is needed to induce lymphoneogenesis and TLS formation [1,13,15].

Stromal elements are particularly important for TLS development in the tumour setting. Fibroblastic reticular cell-like populations and cancer-associated fibroblasts (CAFs) have the potential to gain LTo properties and contribute to chemokine production and the structural support of TLSs. Furthermore, endothelial remodelling and the generation of specialised perivascular niches promote lymphocyte recruitment and the formation of high endothelial venules (HEVs), thereby further enhancing lymphoid neogenesis inside tumours [1,13,16].

Notably, TLSs have dual roles—pro-tumourigenic and anti-tumourigenic—depending on the composition of immune cells in the tissue [1,13]. Mature TLSs containing germinal centres (GCs), dendritic cells (DCs), and T and B cells are associated with improved patient survival and enhanced responses to immunotherapy. In contrast, TLSs with the presence of regulatory T cells (Treg, FOXP3+) or immature HEVs are associated with poor immune infiltration of the tumour and increased metastatic potential. Moreover, intratumoural B cells may contribute to tumour growth and progression through the production of immunosuppressive cytokines, such as IL-10 [14,15,16].

### 3.2. Structural Maturation of TLSs (From Aggregates to Germinal Centre TLSs)

There are three tiers of organisational structure for TLSs, which relate to the stages of lymphoid neogenesis. The initial stage, referred to as early TLSs (E-TLSs), is characterised by dense aggregates of B (CD20+) and T (CD3+) lymphocytes that do not contain FDC networks, HEVs, or GCs and are negative for both CD21 and CD23 expression. These structures are frequently detected during the early stages of tumourigenesis. E-TLSs also contain Tregs and immunosuppressive macrophages that help to establish a tolerogenic microenvironment. The presence of E-TLSs in a tumour is related to both a poorer patient prognosis and reduced responsiveness to treatment with immune checkpoint inhibitors (ICIs). Finally, since E-TLSs represent a transitional developmental stage, their specific function in anti-tumour immunity is still unclear [1,5,17,18].

The next organisation stage is the primary follicle-like TLS (PFL-TLS), which represents partial compartmentalisation into B-cell and T-cell areas. These dense lymphocyte aggregates create a CD21+ FDC network but lack CD23 expression. HEVs may be present but are often irregular or poorly developed. Therefore, only a limited amount of B-cell class switching occurs within PFL-TLSs, and they do not facilitate the maturation of high-affinity antibodies. In addition, tumours that contain PFL-TLSs tend to display transient anti-tumour immune responses [1,5,17,18].

The last stage, corresponding to TLS maturity, is secondary follicle-like TLSs (SFL-TLSs). They display a highly structured architecture, including compartments containing CXCR5+ B-cell follicles, CCR7+ T-cell zones, active GCs, CD21+CD23+ FDC networks, and HEVs. Functionally, the SFL-TLS shares key features with SLOs, particularly the ability to support the activation of GC-dependent B cells to produce high-affinity antibodies targeting tumour antigens. In addition to promoting humoral immune responses, SFL-TLSs also promote effector T-cell and NK-cell responses. In many cancer types, the detection of SFL-TLSs is related to a positive clinical prognosis and enhanced responsiveness to ICIs, highlighting its status as the most biologically active TLS subtype [1,5,17,18].

Some researchers have developed an alternative TLS classification into immature and mature stages based on positive CD23 staining observed in immunohistochemistry (IHC) or immunofluorescence (IF) [1]. The mature TLS was defined by the presence of CD23+ FDCs, BCL6+ GC B cells, and PNAd+ HEVs, while immature TLSs only contained CD20+ B cells (Meylan et al.) [1,19]. In addition, Ahn et al. described the upregulation of 13 proteins (CYRIA, ENG, GPI, HLA-A, LIMA1, LRBA, LST1, MCAM, MGLL, NID1, NME2, PIK3R1, STARD7) in the mature TLS compared to the immature TLS, suggesting their potential as biomarkers of TLS maturation [1,20].

Immature TLSs may also trigger effective immune responses, but these often depend on T-cell exhaustion, inflammatory signalling, or the immunosuppressive nature of the TME. Interestingly, in immature TLSs, B cells tend to be less numerous and usually produce antibodies with limited affinity. In contrast, mature TLSs contain BCL6+ B cells within GCs, where the selective activation and expansion of specific B-cell clones occur, promoting immunoglobulin class switching and somatic hypermutation. As a result, the antibodies produced exhibit high affinity and demonstrate more effective immune function [1].

The organisation of T and B lymphocytes into separate compartments depends on increased chemokine secretion. Notably, the CCL19-CCR7 or CCL21-CCR7 axis is essential for guiding T-cell zones, while the CXCL13-CXCR5 pathway plays a similar role for B-cell areas [18]. Studies indicate that HEVs and DCs are present throughout all TLS maturity stages, as well as in unorganised regions rich in lymphocytes, suggesting that these components might have a meaningful role in TLS development [17].

Thus, TLS maturation provides the structural basis for functional interpretation, but maturity alone does not fully define TLS function without spatial and immune context information.

### 3.3. B-Cell Immunity Within TLSs

Mature B cells in the TME display various degrees of differentiation, including phenotypes that are characteristic of the marginal zone, germinal centre, and extrafollicular compartments. The activation of mature B cells in peripheral lymphoid tissues is associated with or occurs independently of T cells. TLSs provide a local niche for B-cell activation, clonal expansion, and differentiation toward plasma cell and memory cell phenotypes. However, the quality of this response depends strongly on TLS maturation and the immune context [12,14,17,21,22].

In addition to GCs, B-cell activation also occurs through extrafollicular pathways. In this case, naive B cells are stimulated in association with FDCs and CD4+ T peripheral helper (Tph) cells. This leads to the production of atypical memory B cells and short-lived plasma cells. The antibodies synthesised under these conditions are characterised by lower affinity and autoreactivity, playing a diminished role in effective anti-tumour immunity [12].

B cells in TLSs also function as professional antigen-presenting cells (APCs). By expressing major histocompatibility complex (MHC) class II molecules and costimulatory molecules like CD80 and CD86, they are able to activate helper and cytotoxic T cells, thereby contributing to the coordination of anti-tumour immunity in the TME [12,14,21,22].

The overall impact of tumour-infiltrating B cells (TIBs) is context-dependent and may be regarded as a “double-edged sword”. Effector-oriented B-cell and plasma cell responses may support anti-tumour immunity through antigen presentation, antibody production, and cooperation with T cells. Conversely, regulatory B-cell phenotypes may suppress effector T-cell and NK-cell activity and promote regulatory immune programs. Therefore, the clinical significance of B-cell infiltration depends not only on B-cell abundance but also on the balance between effector and regulatory B-cell states within the TLS niche [12,17,21,22,23,24].

The functional role of TIBs is closely linked to TLS maturation and the immune context. Mature, GC-containing TLSs more often support coordinated B-cell, plasma cell, and T-cell responses, whereas immature or regulatory immune niches may be associated with ineffective or suppressive humoral activity. Therefore, B-cell infiltration should be interpreted together with TLS maturity, spatial organisation, and the balance between effector and regulatory immune programs [12,14,21,22,25].

In summary, the functional role of B cells in tumours is largely dependent on the context and closely associated with the maturation status of TLSs. Since immature TLSs are often connected with immunosuppressive or ineffective humoral responses, mature TLSs containing GCs are sites of active B-cell differentiation, affinity maturation, and antibody production. Thus, the qualitative status of B-cell organisation in TLSs, rather than only their presence, is a key determinant of their role in anti-tumour immunity.

### 3.4. Cellular Ecosystem of TLSs

#### 3.4.1. Tfh–B-Cell Axis

The T follicular helper (Tfh)–B-cell axis is central to the functional maturation of GC-positive TLSs. Tfh cells, characterised by CXCR5 expression and regulated in part by BCL6, migrate toward B-cell follicles and provide essential help to B cells through CD40L–CD40 interactions, ICOS-dependent costimulation, and IL-21 secretion [5,26,27,28,29]. These signals support germinal centre reactions, including somatic hypermutation, class-switch recombination, affinity maturation, and differentiation toward plasma cell and memory B-cell programs [5,26,29].

Within TLSs, B cells also function as antigen-presenting cells by capturing antigens through the B-cell receptor, processing them, and presenting antigen-derived peptides on MHC class II molecules to CD4+ T cells, including Tfh cells [12,13]. This reciprocal Tfh–B-cell interaction explains why GC-positive TLSs are often interpreted as active sites of local adaptive immune maturation rather than passive lymphoid aggregates [1,12,16].

However, Tfh-associated programs are not universally protective. In selected tumour contexts, including gastric cancer and osteosarcoma, altered or dysfunctional Tfh–B-cell interactions have been associated with immune suppression, impaired B-cell maturation, or poorer clinical features [27,28,29,30]. Therefore, in the TLS-A/B/C framework, Tfh–B-cell activity supports TLS-A interpretation only when it is accompanied by GC activity and an effector-oriented immune context; in contrast, Tfh-rich niches with regulatory B-cell enrichment or other suppressive features may require a TLS-C-like interpretation.

#### 3.4.2. Regulatory T Lymphocytes (Treg)

Treg lymphocytes represent a specialised T-cell subset with the CD4+CD25+FoxP3+ profile that infiltrates tumour-associated TLSs (TA-TLSs) and contributes to the suppression of anti-tumour immune responses in the TME, thereby promoting tumour growth. The presence of Treg cells correlates with unfavourable prognoses in several malignancies, such as non-small-cell lung cancer (NSCLC), where they inhibit T-cell activation [31,32].

In Kras/p53-driven lung adenocarcinoma (LUAD), activated Treg cells restrained DC–T-cell interactions. T-cell proliferation is restored in Treg depletion, resulting in tumour regression. In TLSs, Treg cells frequently express CD62L, a homing molecule that mediates their migration and accumulation in these structures [5,31].

Importantly, Treg cells located outside TLSs may exhibit even greater immunosuppressive potential. These Tregs often demonstrate the overexpression of multiple immune checkpoint proteins (ICPs) and immunosuppressive cytokines, suggesting that non-TLS Tregs may show greater immunosuppressive capacity compared to TLS Tregs [31].

From a therapeutic standpoint, the selective targeting of immunosuppressive components in TA-TLSs (like Treg cells) represents a highly promising approach to improving local anti-tumour immunity without disrupting systemic immune homeostasis [31,32].

Framework relevance: The TLS-associated enrichment of FOXP3+ Tregs may support TLS-C assignment only when it indicates regulatory dominance within the TLS niche and is accompanied by impaired effector activity or other suppressive immune context features.

#### 3.4.3. Regulatory B Lymphocytes (Bregs)

Regulatory B cells (Bregs) represent a small but functionally important B-cell subset with immunosuppressive activity. In tumour-associated immune niches, Bregs may inhibit cytotoxic CD8+ T cells and natural killer cells, promote Treg differentiation, and contribute to local immune tolerance through cytokines such as IL-10, IL-35, and TGF-β, as well as checkpoint-related mechanisms including PD-L1-dependent signalling [5,12,30]. In several malignancies, Breg enrichment has been associated with advanced disease, metastasis, impaired anti-tumour immunity, and poorer clinical outcomes [5,30].

In the TLS framework, Bregs are relevant not simply as isolated cells but as part of a broader regulatory immune context. Their presence may support TLS-C assignment when Breg-like phenotypes are spatially enriched within or around TLSs and coexist with other suppressive features, such as FOXP3+ Tregs, CD163+/CD206+ macrophages, checkpoint-rich programs, or impaired cytotoxic activity. However, Bregs alone should not define TLS-C without evidence that the TLS niche is functionally dominated by regulatory or suppressive immune programs.

Framework relevance: Breg-like phenotypes support TLS-C interpretation when they are spatially enriched within or around TLSs and coexist with other suppressive features, but they should not define TLS-C in isolation.

#### 3.4.4. Plasma Cells: IgG- and IgA-Associated Responses

Plasma cells (PCs) in TLSs show considerable diversity, with the predominance of one particular immunoglobulin class, especially IgG or IgA, depending on the affected tissue and pathological state. Both IgG+ and IgA+ plasma cells can migrate from TLSs to tumour stroma via tracks rich in fibroblasts. This process is controlled in part by the CXCL12-CXCR4 pathway, in which fibroblasts associated with cancer release CXCL12, creating chemokine gradients for attracting plasma cells [12,19].

IgG+ plasma cells commonly predominate in inflamed tumours. For instance, in colorectal liver metastasis (CRLM), the SFL-TLS regions contain IgG+ plasma cells that contribute to anti-tumour antibody activity. In contrast, an abundance of IgA+ plasma cells is observed in TLS-deficient tumours. Generally, the presence of IgG-producing plasma cells correlates with a more favourable prognosis, including prolonged progression-free survival (PFS), particularly during ICI therapy. IgG+ plasma cells in TLSs produce antibodies that opsonise malignant cells, thus initiating anti-tumour immune responses. Additionally, tumour cells can be eliminated directly due to the IgG-mediated activation of antibody-dependent cellular cytotoxicity (ADCC) [5,12,19].

Notably, not all IgG subclasses are biologically equal. In fact, IgG1 and IgG3 are associated with effective anti-tumour responses, including in renal cell carcinoma (RCC), due to their strong interaction with the Fcγ receptors, thus activating effector pathways. However, IgG4 is described as immunosuppressive due to its poor binding capacity for Fcγ receptors. In some malignancies, such as melanoma, prostate cancer, colorectal cancer, and oesophageal cancer, IgG4 can suppress the activity of other antibody subclasses and promote macrophages’ transition to an immunosuppressive M2-like type, contributing to a poorer prognosis [5,12,33].

In contrast, plasma cells producing IgA are typical for mucosal epithelial tumours, like ovarian, colorectal, gastric, and liver cancers. IgA is a key factor in protecting the mucosa from various pathogens and antigens; thus, the presence of IgA+ plasma cells is often associated with chronic inflammation and localised humoral immunity [5,12,34].

However, the prognostic role of IgA+ plasma cells seems to be conditional and, in many malignancies, is connected with poor clinical outcomes. The accumulation of IgA+ plasma cells is associated with adverse impacts on the courses of prostate cancer, oesophageal cancer, hepatocellular carcinoma (HCC), and colorectal cancer (CRC), presumably due to their immunosuppressive potential. These cells may disrupt anti-tumour responses by secreting inhibitory cytokines like interleukin-10 (IL-10) and also by expressing immune checkpoint molecules, including programmed death ligand 1 (PD-L1), leading to T-cell dysfunction in the TME. Regardless, in certain tumour types, such as ovarian cancer, IgA+ plasma cells may play a protective role. In this case, IgA antibodies are capable of interacting with polymeric immunoglobulin receptors (pIgRs) present on tumour cells, sensitising them to T-cell-mediated cytotoxicity [5,12,19,33].

Framework relevance: IgG-dominant plasma cell responses spatially associated with GC-positive TLSs may support TLS-A interpretation, whereas plasma cell programs linked to regulatory cytokines, checkpoint expression, or impaired cytotoxic immunity may support TLS-C-like interpretation only in an appropriate suppressive immune context.

#### 3.4.5. Myeloid Compartment: M2-like Macrophages, Neutrophils, and Dendritic Cells

Myeloid cells, including dendritic cells, macrophages, monocytes, neutrophils, and myeloid-derived suppressor cells (MDSCs), are important components of the tumour microenvironment and may influence tumour progression, immune activation, and immune suppression [35,36,37,38,39,40,41]. However, in the context of TLSs, their roles should be interpreted not only through their general functions in cancer but also through their localisation within or around TLS niches, their phenotypes, and their interactions with lymphoid and stromal compartments [5,10,42].

Among myeloid populations, dendritic cells are the most directly linked to productive TLS function. They participate in antigen presentation, support local T-cell priming, and may help to organise T-cell zones within developing or mature TLSs [38,42]. In particular, conventional type 1 dendritic cells (cDC1) are relevant because of their ability to cross-present tumour antigens and promote cytotoxic T-cell responses [35,38]. In the proposed framework, TLSs enriched in mature dendritic cell niches, especially when accompanied by germinal centre activity, Tfh–B-cell cooperation, and CD8+ T-cell infiltration, may support a TLS-A-like interpretation.

By contrast, macrophages, neutrophils, and MDSC-like populations may contribute to either inflammatory organisation or immune suppression, depending on their functional state and spatial distribution [5,36,37,38,39,40]. Tumour-associated macrophages are highly plastic and may range from pro-inflammatory, anti-tumour phenotypes to M2-like states associated with CD163/CD206 expression, angiogenesis, tissue remodelling, and the suppression of cytotoxic immunity [36,38,39]. Neutrophil-rich or MDSC-like niches may also contribute to local immune suppression, particularly when associated with pro-tumour inflammatory signals, impaired antigen presentation, or T-cell dysfunction [36,37,40]. In TLS assessment, these populations should therefore be interpreted as part of the immune context module rather than as isolated markers. Their enrichment within or around TLSs may support TLS-C-like assignment only when accompanied by regulatory lymphoid features, checkpoint-rich programs, or impaired effector activity [5,43,44].

A major knowledge gap is that most studies still assess myeloid populations at the broader tumour microenvironment level, whereas their precise localisation and function inside TLSs, at TLS borders, or in adjacent stromal compartments remain insufficiently characterised [5,42,43]. Recent evidence suggests that immunosuppressive macrophages may infiltrate TLSs with distinct maturation statuses, indicating that the myeloid composition may differ between immature and mature TLS states [44]. Future studies using multiplex immunohistochemistry, spatial transcriptomics, and spatial proteomics should therefore clarify whether myeloid cells act as organisers of productive TLS-A-like immunity, modulators of transitional TLS-B states, or contributors to suppressive TLS-C niches.

Framework relevance: Mature dendritic cell-rich TLSs may support TLS-A assignment, whereas the TLS-associated enrichment of CD163+/CD206+ macrophages, neutrophils, or MDSC-like programs may support TLS-C assignment only when spatially linked to a regulatory or impaired effector immune context.

## 4. Architectural Heterogeneity and “Functional States” of TLSs

### 4.1. Three Axes of TLSs: Maturity, Localisation, and Functional Context

Binary TLS scoring (present vs. absent) and approaches based only on density fail to capture the biological heterogeneity of TLSs. Therefore, TLSs have shown inconsistent prognostic and predictive associations across solid tumours and clinical settings. We argue that these discrepancies largely reflect the conflation of at least three independent axes: structural maturity, spatial location, and the functional immune context.

The first axis is structural maturity. Across tumours, lymphoid infiltrates range from loose lymphoid aggregates to organised non-germinal centre (non-GC) TLSs and fully mature germinal centre (GC)-positive TLSs [45,46]. Importantly, GC formation is a qualitative switch that reflects coordinated Tfh–B interactions, affinity maturation, and sustained antigen-driven immune activity. Thus, mature GC TLSs often carry a different clinical meaning from immature aggregates or non-GC TLSs [46]. Consequently, density-only TLS scoring conflates distinct maturation states and can obscure true associations.

The second axis is spatial localisation. TLSs may be intratumoural, concentrated at the invasive margin (marginal), or located in peritumoural or parenchymal tissue (among normal cells) [43]. Intratumoural and margin TLSs are more likely to capture direct tumour–immune engagement, whereas peritumoural TLSs may reflect organ-specific background immunity, chronic inflammation, or tissue remodelling processes that are not necessarily tumour-directed [43,46,47,48]. Because region definitions and sampling strategies vary widely across studies, location is a major (often underreported) source of heterogeneity in TLS-associated outcomes [45,46].

The third axis is the functional immune context. The TLS architecture does not map one-to-one with immune functions—structurally similar TLSs can host effector-prone programs (Tfh–B interactions, cytotoxic T cells, activated B cells and plasma cells) or, conversely, regulatory/suppressive programs characterised by the enrichment of regulatory T cells, regulatory B-cell phenotypes, and suppressive myeloid populations. This functional polarisation provides a mechanistic basis for why some tumours with a large number of TLSs exhibit robust anti-tumour immunity, whereas others display attenuated or even adverse immune states despite the presence of organised lymphoid structures [31,49].

**Figure 1:**
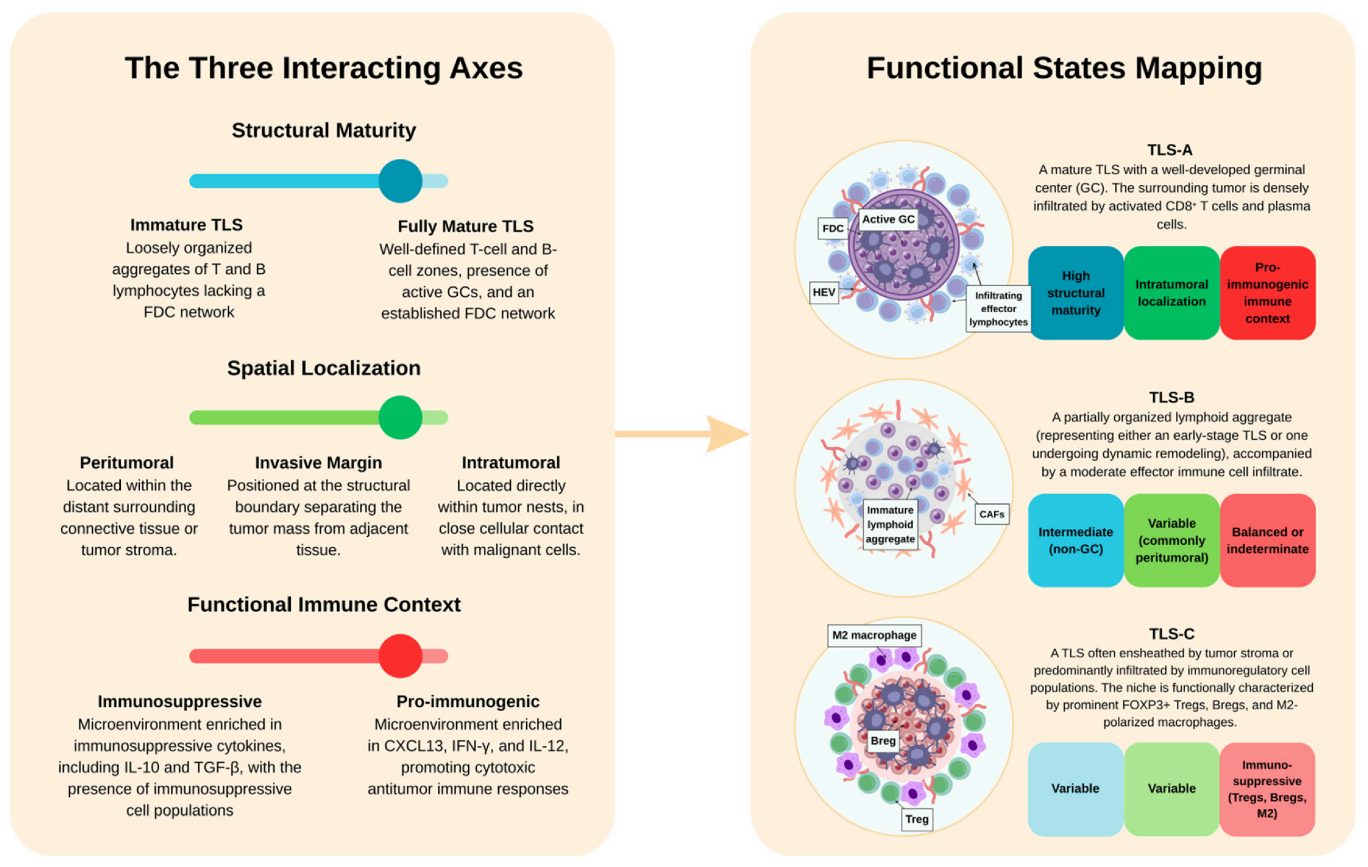
A three-axis model of functional plasticity in TLSs. Schematic representation of the spatial and structural integration of TLSs within the TME. Structural maturity: progression from early, poorly organised T- and B-cell aggregates to fully mature TLSs containing functional GCs. Spatial localisation: tissue compartmentalisation encompassing intratumoural niches, the invasive margin, and peritumoural stromal regions. Functional immune context: polarisation of the cytokine environment and immune cellular composition along a continuum from a pro-immunogenic (effector) to an immunosuppressive (tolerogenic) state. The intersection of these three axes defines three principal functional states: TLS-A (active/pro-immunogenic), TLS-B (transitional/plastic), and TLS-C (immunoregulatory/dysfunctional). The figure was created using Canva Pro web-based version (https://www.canva.com/pro/, accessed July 2026).

Taken together, structural maturity, spatial localisation, and the functional immune context represent sources of TLS heterogeneity (Figure 1). Different combinations of these axes can produce divergent clinical associations under the same TLS-positive label. In the next section, we link these axes to pro- vs. suppressive immune mechanisms and then formalise them into functional TLS states (TLS-A/B/C) to support standardised interpretation.

### 4.2. From Structure to Function: Mechanistic Pathways Shaping TLS Activity

Rather than repeating information about the cellular components and developmental steps described in Section 2, we focus here on how recurrent immune programs emerging within TLSs translate into distinct functional outputs. In brief, TLSs can act as productive hubs of antigen-driven adaptive immunity when maturation and effector programs dominate; they can remain transitional when organisation is incomplete; and they can become immunoregulatory when suppressive programs prevail [1,46,50]. This mechanistic framing provides the rationale for interpreting TLSs beyond morphology and motivates the functional TLS state framework formalised in Section 4.3.

A central mechanism through which TLSs promote anti-tumour immunity is the establishment of an antigen-driven adaptive immune hub within non-lymphoid tissues [51]. When maturation proceeds to GC activity, TLSs support coordinated Tfh–B-cell interactions that enable affinity maturation and the generation of memory and antibody-secreting programs, while simultaneously sustaining effector T-cell activity in the surrounding microenvironment. In this state, TLSs are not merely histologic aggregates but reflect an organised immune microenvironment capable of amplifying and maintaining adaptive responses [17,49,52]. This provides a mechanistic explanation for why mature, GC-positive TLSs often carry different functional and clinical meanings compared to non-GC or immature structures.

A complementary determinant of TLS function is the supporting stromal and recruitment infrastructure that stabilises lymphoid organisation over time [53]. Lymphoid-organising programs can promote compartmentalisation and sustained immune trafficking, enabling TLSs to behave as persistent immune niches rather than transient clusters [51,54]. Recent mechanistic work has shown that specific stromal lineages can actively regulate TLS neogenesis and shape downstream adaptive outputs, providing a possible explanation for why morphologically similar lymphoid aggregates diverge in stability and functional capacity [53].

Not all TLSs mature into durable, GC-active immune hubs. A frequent intermediate outcome is a transitional state in which the TLS architecture is initiated but remains functionally incomplete (e.g., organised yet non-GC), resulting in variable immune output and context-dependent associations [55]. Conversely, TLSs may adopt immunoregulatory programs despite recognisable organisation when regulatory lymphoid or suppressive myeloid pathways dominate. This explains TLS-positive tumours that nevertheless exhibit attenuated or even adverse immune phenotypes [31]. Tumour-based analyses that stratify TLSs by maturation stage support this continuum by demonstrating that immature and mature TLSs can coexist and align with distinct immune microenvironments within the same disease context [44]. Independent clinical observations further show that regulatory cell infiltration within tumour-induced TLSs can be associated with unfavourable outcomes in selected settings, reinforcing that TLSs should be interpreted through the functional context rather than morphology alone [31]. Together, these mechanisms set the stage for the operationalisation of TLSs into functional states in Section 4.3.

### 4.3. Functional TLS States: Conceptual Summary and Assignment Logic

The mechanistic continuum described above motivates a pragmatic, pathology-oriented framework for interpreting TLSs beyond “present/absent” scoring. Rather than introducing a definitive biological taxonomy, we propose three simplified functional TLS states that integrate structural maturity, spatial localisation, and the immune context. These categories should be treated as conceptual and hypothesis-generating rather than as validated diagnostic entities. The definitions below provide the core assignment logic, whereas Table 1 summarises the same states in parallel and Section 7 provides more detailed histopathologic, spatial, immune context, and molecular assessment rules.

#### 4.3.1. TLS-A: Pro-Immunogenic Effector TLSs

**Definition.** TLS-A refers to mature, antigen-driven TLSs in which GC-positive structural maturity is accompanied by an effector-oriented immune context, supporting local adaptive anti-tumour immunity.

**Core assignment requirements.** TLS-A assignment requires evidence of mature or GC-positive TLSs together with effector immune features, such as coordinated Tfh–B-cell activity, CD8+ T-cell enrichment, plasma cell responses, mature dendritic cell niches, HEVs, cytotoxic markers, or supportive TLS/GC/interferon signatures. Intratumoural or invasive-margin localisation may strengthen this interpretation but does not define TLS-A by itself.

**Supportive features.** TLS-A may be supported by chemokine programs associated with lymphoid organisation and B–T-cell cooperation, GC-related transcriptional programs, plasma cell differentiation, antigen presentation, and interferon/cytotoxic immune signatures.

**Key guardrail.** TLS-A should not be assigned on the basis of TLS density, GC positivity, or intratumoural location alone; structural maturity must be concordant with an effector-oriented immune context.

#### 4.3.2. TLS-B: Transitional or Intermediate TLSs

**Definition.** TLS-B refers to organised but incompletely matured TLSs, or TLSs with mixed/intermediate immune features, in which neither a confident TLS-A nor TLS-C assignment can be made.

**Core assignment requirements.** TLS-B requires evidence of an organised TLS architecture beyond a loose lymphoid aggregate, with absent, incomplete, or uncertain GC activity and no clear dominance of either effector or suppressive immune programs.

**Supportive features.** TLS-B may show partial B-/T-cell compartmentalisation, incomplete follicular dendritic cell networks, intermediate lymphoid-organising chemokine signals, or partial TLS-related molecular signatures without robust GC, plasma cell, cytotoxic, or suppressive co-signatures.

**Key guardrail.** TLS-B should not be used as a default label for insufficient data. Cases with inadequate tissue, missing regional annotation, or an insufficient marker panel should be reported as low-confidence or unclassifiable rather than automatically classified as TLS-B.

#### 4.3.3. TLS-C: Immunoregulatory or Suppressive TLS Niches

**Definition.** TLS-C refers to organised TLS niches in which regulatory or suppressive immune programs dominate, resulting in attenuated effective anti-tumour immunity despite a recognisable TLS architecture.

**Core assignment requirements.** TLS-C requires evidence of organised TLSs together with direct or strongly supported regulatory/suppressive dominance within or around the TLS niche, including FOXP3+ Tregs, Breg-like programs, CD163+/CD206+ macrophages, neutrophil-rich or MDSC-like features, checkpoint-rich or IDO1-related suppressive programs, or impaired cytotoxic activity.

**Supportive features.** TLS-C may be supported by spatial or molecular evidence showing that TLS-associated signals coexist with regulatory lymphoid, suppressive myeloid, checkpoint, metabolic, or T-cell dysfunction programs. Peritumoural localisation may raise suspicion in selected tumour contexts but is not sufficient for TLS-C assignment.

**Key guardrail.** TLS-C should not be assigned on the basis of the peritumoural location, immature morphology, TLS density, or even GC positivity alone. GC-positive TLSs may be interpreted as TLS-C-like only when suppressive immune programs dominate within or around the TLS niche.

To avoid redundancy with the detailed assessment framework in Section 7, Table 1 presents these three functional states in parallel using only the most relevant interpretive features.

**Table 1:** Conceptual and operational summary of the proposed TLS functional states.

Feature	TLS-A: Pro-Immunogenic Effector TLSs	TLS-B: Transitional/Intermediate TLSs	TLS-C: Immunoregulatory/Suppressive TLSs
Core meaning	Mature effector TLSs supporting local adaptive anti-tumour immunity.	Organised but incompletely matured or functionally intermediate TLSs.	Organised TLS niche dominated by regulatory or suppressive immune programs.
Structural maturity	Usually GC-positive mature TLSs with follicular architecture [1,5,17,18,19,56,57].	Organised non-GC or partially compartmentalised TLSs [1,5,17,18,19,56,57].	Organised TLSs with or without GC features; maturity alone is insufficient [5,31,32,44,58].
Location modifier	Intratumoural or invasive-margin location may support TLS-A if effector context is present [5,9,43,58,59].	Any location; spatial context modifies interpretation but does not define TLS-B [5,9,43,58,59].	Peritumoural location may support suspicion but does not define TLS-C without suppressive context [5,9,43,58,59].
Dominant immune program	Tfh–B-cell cooperation, GC activity, plasma cell response, dendritic cell niches, CD8+ cytotoxicity [5,18,49,58,60,61,62].	Partial lymphoid organisation, incomplete GC program, or mixed effector/regulatory signals [5,43,44,58,63].	Treg-, Breg-, suppressive myeloid-, checkpoint-, metabolic-, or dysfunction-associated programs [5,31,32,43,44,58,64,65].
Candidate markers	CD20/CD19, CD3/CD8, CD21/CD23, BCL6, Ki-67, PNAd/MECA-79, CD138, DC-LAMP/CD208, granzyme B [1,5,17,18,56,57,58,60].	CD20/CD19 and CD3 with incomplete CD21/CD23, BCL6/Ki-67, CD8, CD138, or PNAd/MECA-79 patterns [1,5,17,18,56,57,58,60].	FOXP3/CD25, Breg-like markers, CD68/CD163/CD206, MPO/CD66b, PD-L1, CTLA-4, LAG-3, TIM-3, IDO1 [5,31,32,43,44,58,64,65].
Typical interpretation	More often favourable; may predict improved response to immune checkpoint blockade in selected settings [49,60,61,66,67,68,69,70].	Context-dependent; may represent a plastic or potentially inducible TLS state [1,42,44,56,58,63,71,72].	Potentially unfavourable or therapy-resistant in selected contexts, but tumour type-dependent [31,32,47,48,71,73,74,75,76].
Main guardrail	Do not assign based on GC positivity or density alone.	Do not use as a default category for insufficient data.	Do not assign based on peritumoural location, immature morphology, or density alone.

Table 1 provides a concise conceptual summary of the TLS-A/B/C framework. Functional assignment should be based on the integrated interpretation of structural maturity, spatial localisation, and the immune context, whereas detailed histopathologic, spatial, immune context, and molecular assessment rules are developed in Section 7. The relationship between these proposed functional states and established TLS maturation classifications is discussed in Section 4.4.

### 4.4. Relationship with Existing TLS Maturation Classifications and Added Value of the Framework

The proposed TLS-A/B/C framework is not intended to replace existing structural maturation schemes, such as E-TLS/PFL-TLS/SFL-TLS or immature/mature TLS classifications [1,5,17,18,19,56,57]. Instead, it builds on them by adding spatial localisation and the immune functional context, which are increasingly recognised as major determinants of TLS interpretation in solid tumours [5,9,43,58]. Therefore, the relationship between conventional TLS maturation categories and TLS-A/B/C should be interpreted as approximate rather than one-to-one. The approximate correspondence between established TLS maturation categories and the proposed functional states is summarised in Table 2.

The added value of the TLS-A/B/C framework is the explicit integration of structural maturity with the location and immune context. Thus, mature TLSs are not automatically TLS-A if regulatory or suppressive programs dominate, and immature or peritumoural TLSs are not automatically TLS-C without evidence of suppressive immune dominance [5,9,31,32,43,44,58]. This functional context axis is intended to explain why morphologically similar TLSs may show favourable, neutral, or adverse clinical associations across tumour types and treatment settings.

## 5. Tumour Microenvironment Archetypes: Context-Dependent Functional States of TLSs

To transfer the TLS-A/B/C framework to biologically recognisable tumour settings, we organise the evidence into four recurrent tumour microenvironment archetypes. These archetypes are not intended as mutually exclusive tumour categories but as interpretative patterns that explain why TLSs may carry favourable, neutral, context-dependent, or adverse clinical significance. In this section, we integrate TLS maturity, spatial localisation, immune context, specimen type, treatment exposure, and tumour stage heterogeneity to illustrate how similar TLS-positive findings may correspond to distinct functional states.

**Figure 2:**
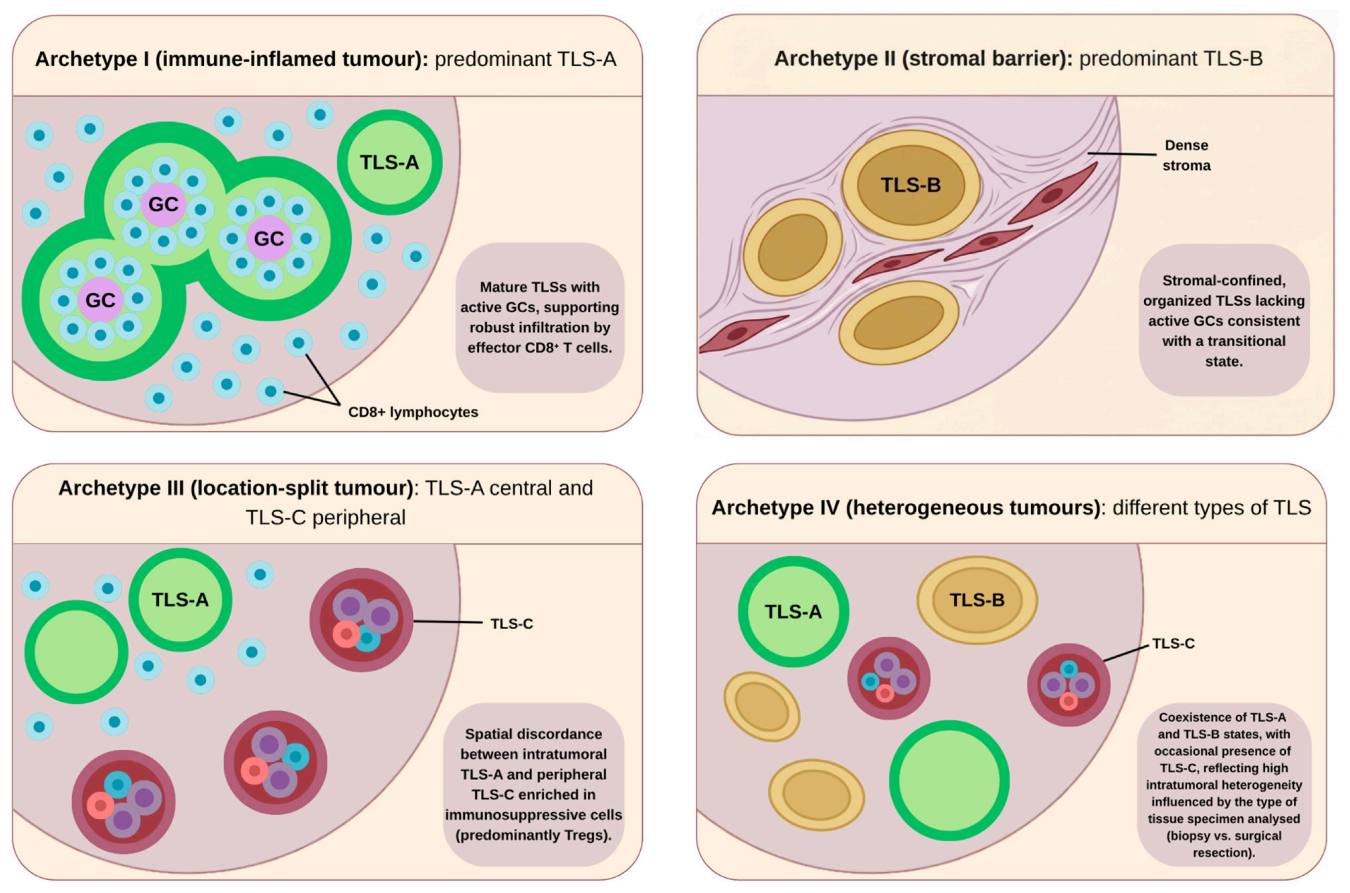
Architectural archetypes of the TME and compartmentalisation of TLS functional states. An integrated conceptual model illustrating four fundamental immune landscape archetypes in solid tumours. Archetype I (immune-inflamed tumour): characterised by dense lymphocytic infiltration and predominance of intratumoural, mature TLS-A with high anti-tumour potential. Archetype II (stromal barrier tumour): characterised by pronounced stromal desmoplasia, spatial restriction of immune cell infiltration to the invasive margin, and predominance of transitional TLS-B. Archetype III (location-split tumour): characterised by spatial dichotomy, with effector TLS-A localised adjacent to malignant cells and immunosuppressive TLS-C preferentially accumulating within the distant peritumoural stroma. Archetype IV (heterogeneous tumour): reflecting a dynamic, mosaic immune landscape with variable potential for therapeutic reprogramming. The figure was created using Canva Pro web-based version (https://www.canva.com/pro/, accessed July 2026).

Importantly, the proposed archetypes of the TME should be interpreted as conceptual and dynamic models rather than as fixed biological categories. Individual tumours may evolve from one archetype to another as the disease progresses or in response to systemic therapy, particularly immunotherapy. Furthermore, significant intratumoural heterogeneity may result in the coexistence of multiple TLS functional states and archetypal patterns in different regions of the same tumour. Therefore, the proposed archetypes are intended to serve as interpretive models to facilitate biological understanding, rather than as mutually exclusive classifications of tumours (Figure 2).

### 5.1. Archetype I: Immune-Inflamed Tumours with Predominant Pro-Immunogenic TLSs (TLS-A)

Immune-inflamed tumours are characterised by the abundant infiltration of effector immune cells, active antigen presentation, and interferon-driven inflammatory signalling [77,78]. Within the proposed framework, this archetype represents the prototypical setting in which mature GC-positive TLSs are expected to correspond to the TLS-A functional state, provided that structural maturity is accompanied by an effector immune context [1,18,79].

This immunological context is supported by coordinated interferon signalling, effective antigen presentation, and chemokine programs, including CXCL12, CXCL13, and CCL19/21. They promote TLS maturation and the duration of adaptive immune responses [1,80,81,82,83]. Mature TLSs containing GC cells are typically located within the tumour or at its invasive margin, where they function as local immune centres supporting antigen presentation and coordinated B- and T-cell responses [18,60,84]. Consequently, TLS-A should be seen as both an indicator and active generator of an immune response.

From a mechanistic point of view, TLS-A is driven by coordinated interactions between Tfh cells and B cells, which sustain GC activity, affinity maturation, plasma cell differentiation, and the generation of memory B cells, while simultaneously supporting the local activation and maintenance of CD8+ T cells in the TME [80,82,85]. Clonal expansion and plasma cell differentiation in mature TLSs have also been associated with a better response to ICBs, further confirming the biological significance of this functional state [86]. In this context, TLS-A represents a dynamic niche selecting and amplifying B- and T-cell clones, integrating the maturation of the humoral response with an effective effector response, and determining the tumour’s susceptibility to immunotherapy.

Multiple studies on the clinical effects of the TLS have been conducted. They conclusively report that mTLSs are associated with better prognoses in various solid tumours, being a favourable biomarker for a better response to immunotherapy [52]. A 2022 study by Brunet et al. [87] showed that mTLSs are a useful biomarker, helping to foresee a response to immunotherapy in patients with non-small-cell lung cancer [87]. Moreover, other studies highlight their importance as biomarkers, correlating with better OS and a more efficient treatment response [88]. However, corticosteroids disturb their maturation and prognostic value [17]. Another 2020 study by Cabrita et al. [49] revealed that the presence of TLSs favours an efficient immune response in melanoma and can be a biomarker for better OS, as well as the response to immune checkpoint inhibitors (ICIs) [49]. More studies support this thesis. A better response to anti-PD1 immunotherapy in renal cancer could be found when there was a high number of TLSs [66,67]. Furthermore, it has been proven that, in renal cancer, mTLSs enhance the local immune response, leading to improved OS and a better treatment response [19]. At the same time, a 2024 study by Gil-Jimenez et al. [89] found that, in urothelial cancer, the efficacy of immunotherapy relies more on the spatial relationships of immune cells. The close proximity of CD8+ T cells and macrophages to tumour cells favours treatment response [89]. Therefore, the presence of mTLSs in the TME is an independent biomarker for a better response and OS in patients treated with ICIs, regardless of PD-L1 expression and the number of CD8+ T cells [61,68]. Studies on the impact of TLSs on CRC have shown that both the presence of mTLSs and their high density can be linked to better survival and a lower risk of tumour recurrence. At the same time, a lack of mTLSs or their low number, especially alongside high Ki-67, is an unfavourable factor [69,84].

In summary, TLS-A represents the most consistently pro-immunogenic TLS state. In immune-inflamed tumours, mature GC-positive TLSs may act both as biomarkers and active organisers of local anti-tumour immunity, supporting improved survival and enhanced responsiveness to immune checkpoint blockade. However, their clinical interpretation should remain dependent on maturity, the spatial context, and effector immune polarisation rather than the TLS density alone.

### 5.2. Archetype II: Stromal Barrier/Immune-Excluded Tumours with Inducible or Transitional TLSs (TLS-B → TLS-A)

Tumours with a stromal barrier or that are immunologically excluded are characterised by a dense desmoplastic stroma, impaired antigen presentation, and the presence of immunosuppressive cell populations that limit the effective influx of lymphocytes into the tumour site [5,90]. Under such conditions, TLSs may form but often remain immature or incompletely functional, which, in the proposed model, corresponds to the transitional TLS-B state.

In this setting, TLSs are not necessarily absent, but they more commonly occur as intermediate structures with limited functionality. In addition to lymphoid aggregates and GC-containing TLSs, an intermediate subgroup can be identified in which TLSs are structurally organised but lack the functionality of GCs [56]. We propose to classify these structures as TLS-B, defined as organised TLSs that have begun to form a lymphatic structure but do not show evidence of a fully mature GC program. They may occur both in the peritumoural regions and within the tumour centre. Although spatial localisation is not itself defining for TLS-B, such structures frequently align with peritumoural regions [5,58]. This model is supported by studies showing that TLSs may arise early during tumour development yet remain functionally immature. In early-stage liver cancer, TLSs were almost exclusively immature, lacking the morphologically mature follicular features. Moreover, they coexisted with inhibitory and immunosuppressive programs, indicating that local immune activation does not necessarily translate into effective tumour control [71].

Importantly, TLS maturation should not be regarded as either an automatic or spontaneous process. Even after lymphoid organisation has been initiated, the transition toward fully mature TLSs containing GCs may remain incomplete. In this context, Posch et al. showed that, in tumours with a low TLS density, the process of lymphoid organisation may be initiated but does not achieve full maturation, resulting in the persistence of early-stage TLSs rather than the development of fully mature GC-containing structures [72]. In addition to the follicular architecture alone, the functional efficiency of the TLS also depends on the organisation of dendritic cell niches, as these cells are the primary antigen-presenting cells that coordinate adaptive immune responses. In this context, incomplete TLS maturation may indicate not only a lack of GC activity but also the insufficient consolidation of local interactions between antigen-presenting cells and T cells [42].

The biological fate of TLS-B appears to remain plastic. Mature TLSs are associated with Th17-type immune programs, whereas immature TLSs more frequently co-occur with CD163+ macrophages and other immunosuppressive features [63]. Furthermore, immature TLSs may promote differentiation toward regulatory B-cell phenotypes, which represents a potential mechanism leading to the transition to the TLS-C state [91].

CXCL13 signalling appears to be one of the main molecular pathways regulating this transition. Progressive changes in the cellular source of CXCL13 during lymphoid follicle development suggest that sustained CXCL13-dependent signalling contributes to the maturation of the GC and the transformation of TLS-B-like structures toward more mature, TLS-A-like states [14].

Given the clinical significance of TLSs, the available evidence suggests that the formation of mature TLSs can be induced by a variety of therapeutic interventions, including vaccination, immunotherapy, chemotherapy, radiotherapy, and cytokine delivery approaches. For this reason, TLSs should be viewed not as a static histological finding but as inducible immune structures whose maturation states may remain biologically plastic under appropriate therapeutic conditions [1].

This plasticity is central to the TLS-B concept: intermediate TLSs should not be interpreted as biologically inert structures but as potentially convertible immune niches whose final orientation depends on stromal, inflammatory, antigenic, and therapeutic cues.

### 5.3. Archetype III: Location-Split Tumours—Spatially Divergent TLS Function and the Risk of Suppressive TLS-C-like Niches

TLSs can be found not only intratumourally but also in the invasive margin and peritumoural region in location-split tumours. Their spatial distribution within the TME is highly heterogeneous and functionally relevant. Therefore, the role of TLSs should be discussed not solely based on their presence or maturity but also on their spatial location in the TME [59,92]. The difference in function is an effect of the distinct inflammatory profile, composition, and antigen availability in particular parts of the tumour [93]. The intratumoural zone is usually rich in tumoural antigens and IFN signals, supporting the activation of effector T cells, the Tfh–B pathway, and the formation of GCs. In the invasive margin, tumoural and stromal signals are combined, leading to mixed cell phenotypes and functions. In contrast, the peritumoural zone is similar to chronic inflammation with the recruitment of myeloid cells of regulatory function. These significant differences affect cytokine and chemokine profiles, as well as cell interactions, further determining the local organisation and function of the immune response [5,48,73]. Nevertheless, the literature often unifies the TLS, leading to heterogeneous data, false results, and mixed information. Moreover, analysing all TLSs as one archetype may neglect real functional differences between TLS-A, TLS-B, and TLS-C. Therefore, location-split tumours should not be interpreted through TLS presence alone. Instead, they illustrate how intratumoural, invasive-margin, and peritumoural TLSs may occupy distinct antigenic and inflammatory niches, leading to divergent functional states. In this archetype, TLS-C-like interpretation should be considered only when peritumoural or compartmentalised TLSs are accompanied by regulatory lymphoid, suppressive myeloid, or checkpoint-dominated immune features.

The difference in TLS function in archetype III is based on the varied access to the tumoural antigen, inflammation signals, and cell composition between intratumoural and peritumoural environments [5]. Accumulating evidence highlights that intratumoural TLSs are more often connected to an active anti-tumoural response, the higher infiltration of T and B cells, and visual Th1 and Th17 pro-inflammatory polarisation. They are more mature, associated with improved survival, and have developed HEVs. Moreover, the frequency of Tfh–B cells is higher in intratumoural than peritumoural TLSs [58,94,95]. In contrast, peritumoural TLSs emerge as a result of chronic antigen stimulation and inflammation, which does not have to correlate with the tumour itself. They can appear after organ damage or fibrosis processes, partially independent of the anti-tumour response. The peritumoural zone is associated with the presence of distinct cell populations, including neutrophils and myeloid cells with regulatory potential [48]. In the peritumoural zone, the key role is played by stromal cells—fibroblastic reticular cells, dendritic cells, and endothelial cells. Together, they organise the migration of lymphocytes to TLSs. T cells such as CD4+-type Tfh are also important in this zone, as they initiate the formation of GCs and the maturation of TLSs via the secretion of CXCL13 and interaction with dendritic and B cells. In such an environment, the Tfh–B pathway may be directed toward autoantigens, leading to GC reactions of limited anti-tumoural specificity [73]. In peritumoural TLSs, B cells may differentiate into plasma cells, producing anti-tumoural antibodies, or, in less mature TLSs, adopt a regulatory phenotype, promoting immunosuppression. Therefore, depending on the cellular component and level of organisation, peritumoural TLSs may favour anti-tumoural responses or, with dominating Tregs, M2 macrophages and immature dendritic cells, promoting tumour progression and invasion. Another factor that may modulate and limit TLS function is the richness of myeloid regulatory cells [8,13]. At the same time, clinical data regarding the role of peritumoural cells remain inconsistent. Peritumoural TLSs may be neutral or even have a positive impact on prognosis. On the other hand, in some cases, they can correlate with worse disease courses, highlighting their context-dependent biological function [58]. Therefore, the presence of TLSs in the peritumoural zone should not be interpreted as an immunosuppressive phenotype, i.e., TLS-C, but rather as an enhanced signal of TME heterogeneity and potentially different immune response regulation dependent on the location.

Consequently, during such complex interactions, a functional split within TLS function may occur. Intratumoural TLSs may act as effective niches for an anti-tumour response—i.e., TLS-A—while peritumoural TLSs may present a context-dependent role. Therefore, the key difference is based not on their presence but on their spatial localisation within the antigenic and immunological gradients of the TME.

However, it needs to be highlighted that the peritumoural location should not be automatically linked to TLS-C. In many studies, TLSs located peritumourally show neutral or even positive prognostic importance. At the same time, the presence of peritumoural TLSs should be interpreted as a marker of the inflammatory response rather than a unified immunosuppression marker [96,97].

In selected clinical settings, peritumoural TLSs have been associated with adverse outcomes, but these findings appear tumour type- and context-dependent and should not be generalised as evidence that peritumoural TLSs are intrinsically TLS-C. A 2025 study by Xu et al. [74] reported that the presence of peritumoural TLSs in head and neck squamous cell carcinoma was linked to worse OS and a higher risk of lymph node metastasis. They were noted to be an unfavourable prognostic factor, supporting tumour progression [74]. In a 2025 study by Yu et al. [75], peritumoural TLSs were often connected with highly advanced oesophageal squamous cell carcinoma, suggesting their impact on disease progression and enhanced inflammation around the tumour. At the same time, TLS presence may also be an effect of an active immune response. However, this response does not translate directly into tumour growth control as effectively as intratumoural mTLSs. Therefore, peritumoural TLSs may have an ambivalent biological role, potentially combining tumour progression promotion and the limited efficacy of the anti-tumour response [75]. On the other hand, peritumoural TLSs were also linked with a lower tumour stage and long-term survival in oesophageal cancer in a 2025 study by Zhai et al. [98]. This further highlights the possible dual role of peritumoural TLSs.

A 2022 study by Devi-Marulkar et al. [31] reported that TLSs are a site of activation and differentiation of T cells, which correlated with a Th1 immune response, cytotoxicity, and better OS in non-small-cell lung cancer. Regulatory T (Tregs) cells are also present within these structures and can also be a prognostic marker. A high density of Tregs is associated with worse OS and may weaken the positive effect of TLSs. Therefore, a balance between cytotoxic CD8+ T cells and Tregs may be critical for prognosis [31].

In breast cancer, peritumoural TLSs are linked with an especially unfavourable prognosis. They occur with high density, in multiple peritumoural locations, highlighting more aggressive and advanced stages. The researchers highlight that peritumoural TLSs may reflect an immunosuppressive or dysfunctional TME that promotes disease progression. However, more studies are needed to determine their biological role in breast cancer [47,76].

In the research by Merali et al. (2024) [99], TLSs rich in cytotoxic T cells and B cells were related to better OS and immunotherapy responses. However, TLSs rich in Tregs and Bregs may limit the anti-tumour response and promote further disease progression. Moreover, it has been shown that TLSs enhance the ICI response through support of the local activation of T cells and production of tumour-specific antigens. In pancreatic ductal adenocarcinoma, the presence of intratumoural TLSs is often correlated with better OS and PFS. However, some therapies, such as chemotherapy or steroids, may impair their formation and function [99].

Taken together, these studies support a context-dependent interpretation of peritumoural TLSs. In the proposed framework, a peritumoural location may raise suspicion for a distinct immune niche, but TLS-C-like interpretation requires additional evidence of regulatory or suppressive immune dominance, such as Treg enrichment, Breg-like programs, suppressive myeloid features, checkpoint-rich profiles, or impaired effector activity.

In summary, standardised reporting of TLS location and the integration of spatial data seems to be a vital factor in the precise interpretation of the TLS’s role in tumour biology and treatment response. It needs to be highlighted that TLSs cannot be interpreted without the spatial context, as it is equally important to their maturation. Moreover, TLSs’ spatial heterogeneity leads to different biological functions and prognoses. Therefore, means of detecting and describing TLSs should be unified to avoid further misinformation and provide the best help for patients. They should combine TLS localisation, the maturity level, and the TME context.

### 5.4. Archetype IV: Intermediate and Heterogeneous Tumours—Mixed TLS States and High Sensitivity to Bias

In archetype IV, there is significant heterogeneity among patients with the same disease. For this reason, intermediate and heterogeneous tumours are particularly susceptible to analytical bias. In particular, the interpretation of TLSs depends on the treatment context. Neoadjuvant therapies can significantly affect the TLS density and maturity, thereby modifying or even invalidating the predictive value of TLSs. In some settings, this is associated with a significant reduction in TLS density and maturation, whereas, in others, it promotes the formation of mature tertiary lymphoid structures [90,100].

Importantly, a single tumour may contain mixed TLS states, including mature effector-oriented TLSs, incompletely matured TLSs, and TLS niches enriched in regulatory or suppressive programs. Therefore, TLS heterogeneity should be interpreted compositionally rather than reduced to a single specimen-level TLS-positive or TLS-negative label. This is consistent with recent pan-cancer spatial evidence showing that TLS maturation, location, and state composition may vary within the same tumour specimen [5].

The clinical significance of TLSs is also influenced by the treatment modality. In patients with non-small-cell lung cancer (NSCLC) receiving chemotherapy, no association was demonstrated between TLSs and either treatment response or progression-free survival [87]. In contrast, earlier studies have shown that TLSs are specifically predictive of the response to immune checkpoint blockade [61,101].

The assessment of the presence of TLS structures in a tumour depends largely on the specimen type. According to Wang et al. (2024) [100], TLSs were found in 98.3% of untreated patients with locally advanced rectal cancer who had resection but only 18.1% of those who underwent biopsy before treatment. This suggests that biopsy-based assessment may be related to a high percentage of false-negative classifications and that the specimen type may have a significant impact on the outcome. This suggests that the samples obtained during a biopsy may not fully reflect the spatial distribution of TLSs within the tumour [100].

Methodological differences in the assessment of TLSs add further to this heterogeneity. Within individual studies, TLSs are not always classified using the same maturity criteria, spatial definitions, or sets of markers. For example, in the case of advanced colorectal cancer, a mature TLS has been defined as the organised infiltration of T and B cells with Ki-67-positive proliferating germinal centres, whereas other studies have focused primarily on broader architectural criteria, including the presence of follicular dendritic cells with evidence of a germinal centre reaction. This means that TLS-positive tumours that appear similar at first glance may in fact correspond to biologically distinct conditions [42,69,72].

In summary, these observations indicate that variability in TLS-related findings in intermediate and heterogeneous tumours stems not only from biological diversity but also from methodological inconsistencies. Part of this heterogeneity may already stem from pathological interpretation, as different pathologists do not always identify TLSs in the same way, particularly when the assessment depends on subtle features of maturity or location. For this reason, the standardisation of TLS maturity assessment in routine practice is essential. This provides strong rationale for the framework proposed in Section 7, in which TLSs are described using a more structured and modular approach [57].

Overall, the four archetypes illustrate why TLSs cannot be interpreted as a universal biomarker independently of the tumour context. The same TLS-positive status may reflect mature effector immunity, incomplete lymphoid organisation, suppressive immune polarisation, or sampling-related heterogeneity. This provides the rationale for the functional and modular assessment strategy proposed in the following sections.

## 6. Therapeutic Implications of TLS Functional States

### 6.1. TLS Functional States as Predictive Biomarkers for Immunotherapy and Neoadjuvant Treatments

TLS functional states are beginning to be viewed as potential biomarkers for predicting treatment response. They may prove particularly useful in the context of immune checkpoint inhibitor therapies and neoadjuvant therapy [57,60,61,97,102]. In this context, a shift in the perception of functional states is also necessary; they should not be viewed in binary terms. An increasing number of studies indicate that the clinical value of TLSs does not lie in their mere presence but rather in their functional state and, above all, in their degree of maturity [60,61,102]. Mature TLSs appear to represent the state with the greatest prognostic value; in a landmark multi-cohort analysis conducted by Vanhersecke et al. (2021) [61], the presence of mature TLSs was associated with a better objective response rate, progression-free survival, and overall survival in patients treated with anti-PD-1/PD-L1 therapy, regardless of PD-L1 expression and CD8+ T-cell density. The results of this study highlight the value of TLSs as a biomarker that goes beyond strictly immunological parameters [61].

Recent studies confirm this interpretation. Hao Li et al. (2025) demonstrated in their work that the presence of mature TLSs correlates with better overall survival and responses to ICBs and that tumours lacking mature TLSs do not support local anti-tumour immunity [60]. Mature TLSs are enriched in T cells with stem cell-like properties, as well as differentiating B cells, which together sustain local anti-tumour immunity, whereas immature TLSs are more frequently associated with immunosuppressive immune activity [60,102]. This highlights the potential for using TLS maturity as a biomarker. However, TLSs should not simply be classified as present or absent but rather stratified according to their functional status [60,61,102].

The concepts outlined above take on particular significance in the case of gastrointestinal cancers. In rectal cancer, the detection of TLSs has been shown to predict a better response to neoadjuvant therapy [100]. Similarly, in the case of colorectal adenocarcinoma, a CRC-specific TLS signature has enabled the identification of patients who are more likely to benefit from both neoadjuvant chemotherapy and PD-1 blockade [103]. The findings from the aforementioned studies indicate that mature TLSs are not merely biomarkers of pre-existing immune activation but can serve as tools for assessing prognosis and the response to neoadjuvant treatment [60,61,100,103].

### 6.2. Therapeutic Induction of Pro-Immunogenic TLS States (TLS-A)

The application of treatments utilising TLSs should focus not on increasing the number of lymphoid clusters but on shifting their stage of development toward mature forms [13,15,104,105,106]. TLS-A can be understood as mature, organised clusters capable of developing and sustaining a local anti-tumour response [13,104]. Preclinical evidence suggests that such a shift in the functional state of TLSs can be achieved in several ways, including stromal remodelling, local inflammatory stimulation, oncolytic virotherapy, and conventional chemotherapeutic and radiotherapeutic interventions [13,104,105,106].

Stromal remodelling appears to be the most compelling method of TLS induction. The work of Johansson-Percival et al. (2021) shows that TLS induction is closely linked to the reprogramming of the tumour stroma, particularly through the normalisation of the vascular system, the modulation of fibroblast-related organisational functions, and the activation of chemokine programmes involving LIGHT/LTα/LTβ, TNFα, and CCL/CXCL signalling [104]. This is of particular significance in tumours with dense desmoplasia, one of which is pancreatic ductal adenocarcinoma (PDAC). Francesca R Delvecchio et al. demonstrated, in a mouse model, that the proximity of dendritic cells and B lymphocytes within the organised structure of mature TLSs may facilitate better communication between these cells, which in turn promotes the development of a more effective anti-tumour response. Induced TLSs in this model enhanced the anti-tumour effects of chemotherapy [15].

Another possible route for inducing TLSs is oncolytic virotherapy. The use of oncolytic viruses has a beneficial effect on the release of chemokines. This intensifies local inflammation and helps to transform tumours that weakly activate the inflammatory response into those that activate it strongly [105,106]. This concept has been verified in mouse models. In their study, Meng-Jie Zhang et al. (2025) [106] demonstrated that the oncolytic herpes simplex virus type 1 (oHSV) induces TLS formation and enhances anti-tumour immunity via the CXCL10/CXCR3 axis. It is also worth noting that blocking this axis impaired TLS formation [106].

Recent reviews indicate that chemotherapy, radiotherapy, and chemoradiotherapy promote TLS formation, particularly when used in combination with immunomodulatory interventions that facilitate the recruitment of immune cells and local lymphoid organisation. However, the aim should not be to induce TLSs per se but to promote states corresponding to TLS-A, which favour an anti-tumour response [13,104]. Consequently, future research should focus on quality-oriented TLS induction, transforming poorly organised or absent lymphoid responses into mature, pro-immunogenic TLSs, rather than merely maximising their numbers [13,15,104,105,106].

### 6.3. Avoiding or Reprogramming Adverse or Suppressive TLS States (TLS-B/TLS-C)

TLSs are often associated with a more favourable prognosis and a better response to treatment, although a growing body of evidence suggests that not all TLSs are beneficial. Immature TLSs may, in selected contexts, act as niches that promote tumour persistence, rather than acting as effective sites for anti-tumour immune responses [32,71,73,107]. Experimental studies on lung cancer have shown that tumour-associated TLSs can be infiltrated by regulatory T cells (Tregs), which actively suppress the responses of effector T cells, supporting the concept that some TLSs have an immunosuppressive rather than a protective effect [32,107]. Similarly, in the case of hepatocarcinogenesis, it has been shown that immature TLSs act as micro-niches for tumour progenitor cells, suggesting that these forms, under specific conditions, facilitate tumour initiation, maintenance, or recurrence rather than immune control [73].

The adverse effect of immature TLS forms is also confirmed by data from translational studies in humans. In early-stage liver tumours, immature TLS forms were associated with higher expression of immunosuppressive molecules. This suggests that immature TLSs may contribute to immunosuppression [71]. Recent reviews emphasise that the heterogeneity of TLSs is not limited to their density or anatomical location but extends to their cellular composition, degree of maturity, and regulatory balance, all of which can shift these structures toward anti-tumour immunity or immunosuppression [107].

Given the above, future therapeutic strategies should aim to remodel immature or suppressive TLS configurations rather than simply increase TLS numbers. In the proposed framework, this would correspond to promoting the transition of incompletely matured TLS-B states or suppressive TLS-C-like niches toward mature, effector-oriented TLS-A-like states, where biologically feasible. This concept is more consistent with the functional nature of TLS assessment, because the clinical value of TLSs depends on their quality, organisation, spatial context, and immunological orientation, rather than solely on quantity [32,71,73,107].

## 7. Methodological Approaches to Assessing TLS Functional States and Proposal of a “Functional TLS Score”

### 7.1. Histopathologic Assessment of TLS: From H&E Screening to Standardised Mature TLS Identification

Conventional histopathology remains the first-line approach for TLS assessment because it is feasible in routine tumour specimens and allows the initial recognition of lymphoid aggregates, follicle-like organisation, and GC formation [17,56,57,108]. On haematoxylin and eosin (H&E)- or haematoxylin, eosin, and saffron (HES)-stained sections, TLSs should first be stratified along a structural maturation axis: loose lymphoid aggregates without a clear follicular architecture; organised non-GC TLSs with partial lymphoid compartmentalisation; and mTLSs showing a GC-like morphology [1,17,56,57,108]. This distinction is biologically relevant because GC formation reflects a transition toward coordinated Tfh–B-cell cooperation, B-cell selection, somatic hypermutation, class-switch recombination, and local adaptive immune activity [17,18,43,58]. Standardised pathology approaches suggest that mTLSs can be operationally identified by visible GC formation on H&E/HES or by immunohistochemical evidence of CD23-positive follicular dendritic cell networks [57]. Because functional TLS assignment requires more than structural recognition alone, the proposed framework separates TLS assessment into a limited set of histopathologic, immunohistochemical, spatial, and optional molecular variables. These variables are not intended to represent a mandatory universal panel but rather offer a practical assessment toolbox that can be adapted to the available tissue, laboratory resources, and study design. Table 3 summarises the core variables that may support structural TLS detection, maturity grading, immune context assessment, and optional molecular interpretation.

Importantly, no single marker is sufficient to define a functional TLS state. GC-associated markers support mature TLS identification, but TLS-A assignment requires an effector-oriented immune context. Conversely, suppressive markers support TLS-C only when they indicate regulatory or suppressive dominance within or around an organised TLS niche, rather than nonspecific background inflammation.

### 7.2. Multiplex Immunohistochemistry, Digital Pathology, and Spatial Technologies: Defining the Immune Context Module

Although conventional histopathology and basic immunohistochemistry define the structural backbone of TLS assessment, they do not fully determine whether a given TLS acts as an effector, transitional, or suppressive immune niche. Morphologically similar TLSs may differ substantially in cellular composition, spatial organisation, and immune polarisation; therefore, structural maturity should be complemented by an immune context module [5,9,43,58]. Multiplex immunohistochemistry and multiplex immunofluorescence allow the simultaneous assessment of B-cell, T-cell, dendritic cell, plasma cell, vascular, regulatory, and myeloid compartments within the same tissue section [5,56,59]. In practical terms, an effector-oriented multiplex panel may combine B-cell and T-cell markers such as CD20/CD19, CD3, and CD8 with GC- and Tfh-associated markers including BCL6, Ki-67, CXCR5, ICOS, PD-1, and CD40L; plasma cell markers such as CD138; mature dendritic cell markers such as DC-LAMP/CD208; cytotoxicity-associated markers such as granzyme B; and vascular markers such as PNAd/MECA-79 [5,18,56,58,59,60,62]. Such profiles support TLS-A assignment when they occur within GC-positive TLSs and are spatially coupled to tumour-facing immune activation. Conversely, a suppressive context panel may include FOXP3 and CD25 for regulatory T cells, markers of Breg-like or regulatory B-cell programs where available, CD68/CD163/CD206 for macrophage-rich or M2-like myeloid compartments, MPO or CD66b for neutrophils, and immune checkpoint or metabolic markers such as PD-L1, CTLA-4, LAG-3, TIM-3, and IDO1 [5,31,32,43,44,58]. These features may support TLS-C assignment only when they indicate regulatory or suppressive dominance within or around the TLS niche, rather than merely reflecting background inflammation or a peritumoural location. TLS-B should remain a conservative intermediate category for organised TLSs with incomplete effector maturation or mixed immune context features.

Importantly, functional TLS assessment should not rely only on marker abundance but also on spatial relationships. The biological meaning of a given immune subset depends on whether it is located inside the TLS, at the TLS border, in the tumour nest, at the invasive margin, or in the peritumoural stroma [5,9,59]. Spatially resolved analyses can quantify immune neighbourhoods, cell–cell proximity, the TLS–tumour distance, and compartment-specific immune programs. For example, the proximity of Tfh cells to B-cell follicles, CD8+ T cells to tumour nests, plasma cells to TLS borders, or mature dendritic cells to T-cell areas may support an effector-oriented TLS-A-like configuration. Conversely, the accumulation of FOXP3+ Tregs, Breg-like programs, CD163+/CD206+ macrophages, neutrophils, or checkpoint-positive cells within or around TLSs may suggest a suppressive TLS-C-like niche when accompanied by impaired effector activity [31,32,44,59,60,62].

Digital pathology may further support this process by automating TLS detection, density measurement, maturity grading, regional annotation, marker quantification, and spatial distance analyses; however, such algorithms require standardised TLS definitions and careful biological validation before routine use [5,57,60,70,109]. Spatial transcriptomics and spatial proteomics add another layer by linking the TLS morphology to localised chemokine, interferon, GC-related, exhaustion, myeloid, and suppressive programs while preserving the anatomical context [5,59,60,62]. Thus, multiplex and spatial approaches provide the immune context information required to move from structural TLS recognition toward functional TLS-A/B/C assignment: TLS-A as mature effector TLSs, TLS-B as intermediate or mixed TLSs, and TLS-C as suppressive TLSs, requiring direct evidence of regulatory immune dominance.

### 7.3. Transcriptomic TLS Signatures and Pan-Cancer TLS Scores: Molecular Support for Functional Assignment

Transcriptomic TLS signatures provide an additional molecular layer for evaluating TLSs, particularly in large cohorts, where detailed spatial or multiplex tissue analysis is not always feasible. These signatures usually combine genes reflecting lymphoid organisation, B-cell recruitment, Tfh–B-cell cooperation, GC activity, plasma cell differentiation, antigen presentation, interferon-driven inflammation, cytotoxicity, and immune regulation [5,9,43,58]. Core TLS-associated signals commonly include chemokines and lymphoid-organising molecules such as CXCL13, CCL19, CCL21, CXCL9, CXCL10, CXCL11, LTA, LTB, LTBR, CCR7, and CXCR5; B-cell markers such as MS4A1/CD20, CD19, CD79A, CD79B, and CD40; Tfh/GC-related markers such as BCL6, ICOS, PDCD1/PD-1, IL21, CD40LG, and AICDA; and plasma cell or antibody-associated genes such as MZB1, JCHAIN, XBP1, IGHG1, and IGHA1 [5,9,18,43,58,103,110]. In addition, effector TLS programs may include antigen presentation and inflammatory genes, including HLA-DRA, HLA-DRB1, CD74, IFNG, STAT1, CXCL9, CXCL10, GZMB, and PRF1, whereas suppressive or TLS-C-like molecular contexts may be supported by regulatory or myeloid-associated markers such as FOXP3, IL10, TGFB1, IDO1, CD274/PD-L1, CTLA4, LAG3, HAVCR2/TIM-3, MRC1/CD206, CD163, VSIG4, and ARG1 [5,9,31,32,43,44,58,64,65]. For example, in colorectal cancer, one TLS-related model included genes such as CCL19/CCL21, CD8A, IGHG1, MRC1, SELL, VSIG4, and PRRX1, while PRRX1, SELL, and VSIG4 were highlighted as important hub genes linked to immune infiltration and tumour microenvironmental regulation [110]. In advanced non-small-cell lung cancer, a TLS-derived gene signature was reported to predict the response to chemoimmunotherapy and correlated with PD-L1/CD274 expression and “hot” immune phenotypes [65]. In the proposed framework, TLS-A would be expected to align with strong TLS-related chemokine, GC/Tfh/B-cell, plasma cell, antigen presentation, and interferon/cytotoxic signatures. TLS-B may show intermediate lymphoid-organising signals, such as CXCL13–CCL19/CCL21 activity, without robust GC, plasma cell, or cytotoxic co-signatures. TLS-C should not be inferred from a TLS-high signature alone but rather from the coexistence of TLS-associated signals with regulatory lymphoid, suppressive myeloid, checkpoint, metabolic, or T-cell dysfunction programs, ideally supported by spatial evidence that these programs localise to the TLS niche. However, transcriptomic TLS scores have important limitations: bulk RNA sequencing does not directly show whether immune cells are organised into true TLSs or dispersed throughout the tumour microenvironment, cannot reliably distinguish intratumoural from peritumoural TLSs, and may be affected by the tumour purity, stromal content, necrosis, sampling region, and treatment timing [5,9,60,65]. Therefore, transcriptomic signatures should be interpreted as an optional molecular module of the proposed functional TLS score—useful for supporting and scaling TLS assessment but insufficient to replace histopathologic maturity grading and spatial immune context evaluation.

### 7.4. Toward a Pathology-Oriented Functional TLS Scoring Framework: Modular Architecture and Assignment Rules

The preceding sections indicate that TLS assessment cannot rely on a single parameter, such as presence, density, or maturity, alone. Histopathology provides the structural backbone of TLS evaluation; multiplex and spatial approaches define the immune context module; and transcriptomic signatures may provide additional molecular support [5,9,43,57,58]. However, these dimensions are often analysed separately, which may contribute to inconsistent clinical interpretation across tumour types, specimen types, and treatment settings [5,9,43,58]. Therefore, we propose a pathology-oriented Functional TLS Score as a structured framework integrating structural maturity, the TLS burden, spatial localisation, the immune cell composition, and optional molecular signatures. This framework is designed to support translational studies, harmonised reporting, and future validation by integrating structural, spatial, immune context, and optional molecular information.

The rationale for a modular score is that each layer of TLS assessment captures a different biological dimension:

1. The structural module distinguishes loose lymphoid aggregates, organised non-GC TLSs, and GC-positive mTLSs, thereby identifying whether lymphoid neogenesis has progressed toward follicle-like maturation [1,17,56,57,108];
2. The density module quantifies the TLS burden via the TLS count, TLS density, TLS area, or maturity-weighted scores, which have shown prognostic relevance in gastrointestinal and colorectal cancers but do not independently define function [70,108,109];
3. The spatial module records whether TLSs are intratumoural, located at the invasive margin, or peritumoural and should be interpreted as a modifier rather than as a standalone functional criterion [5,9,43,58];
4. The immune context module evaluates the balance between effector components, such as CD8+ T cells, T follicular helper cells, GC B cells, plasma cells, mature dendritic cells, and high endothelial venules, and suppressive components, such as FOXP3+ regulatory T cells, regulatory B-cell phenotypes, CD163+/CD206+ macrophages, neutrophils, immune checkpoint expression, or IDO1-related metabolic suppression [5,18,31,32,43,44,58,59,60,62];
5. Finally, the molecular module may support assignment using TLS-related, GC/Tfh/B-cell, interferon/cytotoxic or suppressive gene signatures, but should not replace histopathologic and spatial validation [64,65,103,110].

For practical use, the proposed Functional TLS Score should be applied as a tiered framework rather than as a single mandatory marker panel. This approach reflects the fact that TLS assessment may be performed in routine pathology laboratories with limited immunohistochemistry, in intermediate settings with expanded marker panels, or in research-intensive settings using multiplex, spatial, digital, or molecular technologies. Accordingly, low-resource assessment can identify candidate TLSs and estimate structural maturity, whereas higher-confidence functional assignment requires the integration of structural maturity with localisation, the immune context, and, when available, molecular support. Table 4 summarises this tiered implementation strategy.

The final output of the proposed framework should be a functional assignment as TLS-A, TLS-B, TLS-C, mixed TLS state specimens, or unclassifiable TLSs, rather than a single universal numerical score. TLS-A should be assigned when mature or GC-positive TLSs are accompanied by an effector-oriented immune context, including Tfh–B-cell cooperation, CD8+ T-cell enrichment, plasma cell responses, mature dendritic cell niches, HEVs, cytotoxic markers, or supportive TLS/GC/interferon signatures [5,17,18,57,58,60,64,65,103,110]. TLS-B should be reserved for organised but incompletely matured TLSs with mixed or intermediate immune features, provided that sufficient tissue and marker data are available [1,5,17,56,58,77]. TLS-C should be assigned only when organised TLSs are spatially associated with regulatory or suppressive dominance, including FOXP3+ Tregs, Breg-like programs, CD163+/CD206+ macrophages, neutrophils, checkpoint-rich or IDO1-related suppressive programs, or impaired cytotoxic activity [5,31,32,43,44,58,64,65].

Peritumoural localisation, the TLS density, an immature morphology, or GC positivity alone should not determine functional assignment. Thus, GC-associated maturity should not automatically override immune context information, and GC-positive TLSs may still be interpreted as TLS-C-like if suppressive immune programs dominate within or around the TLS niche [31,32,44,71]. Conversely, immature or peritumoural TLSs should not be classified as TLS-C without direct evidence of suppressive immune dominance. Cases with insufficient tissue, inadequate marker panels, missing regional annotation, or discordant results should be reported as low-confidence or unclassifiable rather than forced into one of the three functional states.

To improve the interpretability and reproducibility, each TLS-A/B/C assignment should ideally be accompanied by a confidence level reflecting the completeness and concordance of the available data. High-confidence TLS-A requires GC-positive maturity together with effector immune context features and, when available, supportive spatial or molecular findings. High-confidence TLS-C requires organised TLSs with direct evidence of regulatory or suppressive dominance. Cases with insufficient tissue, inadequate marker panels, missing regional annotation, or discordant results should be designated as unclassifiable or low-confidence rather than forced into one of the three functional states. Future studies should test tumour-specific reproducibility, interobserver agreement, and prospective associations with clinical outcomes [5,9,57,64,65,70,103,108,109,110].

To facilitate the reproducible use of this framework across tumour types and study designs, we propose a minimum reporting checklist for TLS assessment, summarising the essential variables that should accompany any TLS-A/B/C assignment in translational or pathology-oriented studies (Table 5).

### 7.5. Handling Intratumoural Heterogeneity and Mixed TLS State Assignments

A practical limitation of any TLS classification framework is that multiple TLS states may coexist within the same tumour specimen. Therefore, TLS-A/B/C assignment should preferably be performed at the level of individual TLSs or spatially annotated tumour regions, rather than imposed as a single global label for the entire specimen. Recent pan-cancer spatial profiling by Cho et al. showed that TLSs’ spatial organisation, maturation, and state composition varied across and within tumour specimens, supporting the need for maturation-aware and composition-aware TLS assessment [111].

At the specimen level, we propose reporting the predominant TLS state whenever one category clearly dominates—for example, as TLS-A-predominant, TLS-B-predominant, or TLS-C-predominant—with secondary TLS components described separately when relevant. The designation “mixed TLS state specimen” should be reserved for cases in which no single TLS state clearly predominates or in which coexisting TLS states are sufficiently represented to affect biological interpretation. Whenever possible, the density, proportion, and location of each TLS state should be documented separately. Such coexistence may reflect a biological maturation continuum, spatially divergent immune niches, treatment-related remodelling, or sampling-related limitations.

### 7.6. Illustrative Application to Published Studies

Because the proposed framework is not yet validated and does not define universal thresholds, the following examples are intended only to illustrate how TLS-A/B/C assignment could be approached using published data.

Example 1: mature TLSs and immunotherapy response. In the multi-cohort analysis by Vanhersecke et al. [61], mature TLSs predicted immune checkpoint inhibitor efficacy in solid tumours independently of PD-L1 expression and CD8+ T-cell density. Within the proposed framework, GC-positive mature TLSs, associated with an improved response to immune checkpoint blockade, would represent structural candidates for TLS-A. However, TLS-A assignment would still require concordance with an effector-oriented immune context, such as evidence of GC activity, organised B- and T-cell areas, mature dendritic cell niches, or cytotoxic immune infiltration. This example illustrates how the framework extends a maturity-based observation by requiring functional context confirmation.

Example 2: immature or regulatory TLSs in early liver lesions. Meylan et al. [82] reported that early hepatic lesions displayed immature TLSs and elevated expression of immune inhibitory and immunosuppressive molecules. In the proposed framework, immature TLSs without convincing GC activity would initially correspond to TLS-B candidates. If spatial or molecular assessment confirms regulatory or suppressive dominance within the TLS niche, such lesions may instead be interpreted as TLS-C-like. This example illustrates why immature TLSs should not automatically be considered non-functional and why a suppressive immune context is required before assigning TLS-C.

## 8. Future Validation of the Proposed TLS Framework

The proposed TLS-A/B/C model requires, primarily, prospective validation in large and homogeneous oncology cohorts, using standard histopathological assessment criteria. In the first stage, it would be necessary to assess the inter-pathologist reproducibility of the classification and its consistency with classical TLS maturity markers. Secondly, the use of techniques such as multiplex immunohistochemistry/immunofluorescence, spatial transcriptomics, or spatial proteomics would allow us to verify whether the proposed TLS states actually correspond to distinct cellular and molecular programs [112,113]. Moreover, it would specify whether they reflect the specific spatial organisation of the TME. The next step should be to use digital pathology tools and machine learning algorithms to automatically classify TLS states based on morphological, spatial, and immunophenotypic features. This could reduce the subjectivity of the assessment and increase its reproducibility [114]. Final validation would require prospective studies in which assigned TLS states are correlated with the response to immunotherapy, patient survival, and other clinical outcomes, helping to determine whether the proposed model truly reflects biologically and clinically distinct TLS phenotypes.

## 9. Knowledge Gaps and Future Directions

In our work, we have proposed a conception of the functional states of TLSs, addressing the inconsistencies in the literature findings about their tumoural role. We have moved from the present approach, which is mostly based on quantity parameters, to a more complex interpretation of TLSs. Taking into consideration TLS maturity, location, and the immune context allows reasoning for their variable roles reported in the studies. In the proposed approach, TLS diversity mirrors their functional heterogeneity, which may help to fill the knowledge gap and succeed in facilitating TLS understanding [5].

A more critical interpretation of TLSs is also required because TLSs are not universally beneficial across tumour types or treatment settings. Peritumoural TLSs have been associated with adverse outcomes in some cancers, including breast cancer and hepatocellular carcinoma, but neutral or favourable associations have also been reported in selected contexts [47,48,75,76,96,97,98]. Similarly, B-cell and plasma cell subsets may support anti-tumour immunity when linked to GC activity, antigen presentation, and IgG-dominant effector responses, but they may also contribute to regulatory or ineffective immune programs depending on the phenotype, immunoglobulin class, and spatial organisation [12,19,25,30,33,34]. Finally, some studies indicate that TLSs may have limited or no predictive value outside specific therapeutic contexts, particularly when TLS assessment does not indicate their maturity, location, and immune context [9,87]. These exceptions reinforce the need for functional rather than purely quantitative TLS interpretation.

Nevertheless, the proposed TLS-A/B/C states should be interpreted as functional patterns along a continuum rather than as sharply separated categories. In clinical practice and translational studies, different TLS states may coexist within the same tumour microenvironment, reflecting intratumoural spatial heterogeneity, different stages of TLS maturation, local antigenic gradients, treatment-related remodelling, or technical sampling limitations. Therefore, mixed TLS state specimens should not automatically be regarded as misclassified cases. Instead, they should be reported compositionally, with attention to the proportion, location, maturity, and immune context of each TLS state. A key challenge for future studies will be to distinguish the functional orientation of TLSs from static histological snapshots, which may not fully capture dynamic immune remodelling over time [2,3,5].

Another limitation is linked to the lack of standardised methods of TLS assessment, both molecular and histopathological. Differences in maturity, spatial analysis, or tissue sampling make it difficult to compare results across studies. It seems that only the integration of data, which would include spatial imaging, transcriptomic profiling, and cellular population characteristics, will allow for the reliable assignment of TLSs to a specific functional state and their treatment correlation [2,3].

Furthermore, it will be crucial to further research the plasticity of TLSs. The available data suggest that these structures may undergo remodelling in response to changes in the TME, including treatment. This allows the possibility that TLSs are not solely passive markers of the immune response but active components that can be therapeutically modulated. A particularly interesting aspect is the identification of factors promoting the transition from immature or suppressive phenotypes (TLS-B/TLS-C) to the effector phenotype (TLS-A), which could potentially enhance the efficacy of immunotherapy [3,11,12].

On the other hand, it is possible that, in certain contexts, the induction or presence of TLSs may have adverse effects. This concerns mainly immunosuppressive treatments [12]. This highlights the need for a careful approach to the concept of therapeutically inducing TLSs without considering their functional quality. In such scenarios, future strategies should focus not only on increasing the number of TLSs but, above all, on the targeted modulation of their cellular composition and function.

In summary, the proposed model of the functional states of TLSs is a useful interpretational scheme. However, its practical worth will greatly depend on its implementation possibilities in clinical studies. A key set would be the development of standardised, reproducible TLS assessment tools such as integrated indices. Only such an approach will allow for the full exploitation of the potential of TLSs as biomarkers and therapeutic targets in modern oncology.

## 10. Conclusions

Tertiary lymphoid structures should not be interpreted solely as present or absent histological findings. Their biological and clinical significance depends on their structural maturity, spatial localisation, and immune cell composition. Mature germinal centre-positive TLSs are most consistently associated with coordinated anti-tumour immunity and an improved response to immunotherapy, whereas incompletely matured or suppressive TLSs may have neutral, context-dependent, or even adverse significance.

In this review, we propose a pathology-oriented framework that groups TLSs into three simplified functional states: TLS-A, TLS-B, and TLS-C. This model is intended to support the more consistent interpretation and reporting of TLSs across tumour types, while future prospective studies should determine its reproducibility, clinical validity, and predictive utility.

Because the immune microenvironments of tumours are dynamic, the proposed archetypes should be viewed as fluid states that may change over time and vary across different regions of the tumour, rather than as fixed categories of tumours.

Future studies should validate this framework in prospective, tumour-specific cohorts using standardised definitions, reproducible pathology workflows, and spatially resolved immune profiling. Such efforts may help to clarify when TLSs act as biomarkers of effective anti-tumour immunity, when they represent transitional immune niches, and when they may contribute to immunosuppression or therapeutic resistance. Ultimately, functional TLS assessment may support more precise biomarker development and guide future strategies aimed not only at inducing TLSs but at promoting their maturation toward pro-immunogenic states.

## Figures and Tables

**Table 2:** Approximate relationship between existing TLS maturation categories and the proposed functional states.

Existing TLS Category	Approximate Interpretation in the TLS-A/B/C Framework	Key Added Requirement
E-TLS/lymphoid aggregate	Usually unassigned or low-confidence TLS-B candidate	Requires evidence of organised TLS architecture before functional assignment
PFL-TLS/organised non-GC TLS	Usually TLS-B candidate	Immune context determines whether it remains intermediate or shows TLS-C-like features
SFL-TLS/mature GC-positive TLS	Structural candidate for TLS-A	Requires effector-oriented immune context; maturity alone is insufficient
Immature TLS	Usually TLS-B candidate	May support TLS-C-like interpretation if suppressive programs dominate
Mature TLS	Structural candidate for TLS-A	Requires concordance between GC-positive maturity, spatial context, and effector polarisation

**Table 3:** Minimal histopathologic and immunohistochemical markers for structural TLS assessment.

Assessment Domain	Suggested Variables/Markers	Main Purpose	Relevance to TLS-A/B/C Assignment
Initial morphology	H&E/HES	Identification of lymphoid aggregates, follicle-like organisation, and visible germinal centre-like areas [1,17,57,70,108].	Entry point for TLS detection; loose aggregates alone should not be assigned as TLS-A/B/C with high confidence.
B- and T-cell organisation	CD20 or CD19; CD3 ± CD8	Confirmation of B-cell follicles, T-cell zones, and partial compartmentalisation [1,18,57,70].	Supports recognition of organised TLSs; CD8 enrichment may support TLS-A when coupled with effector context.
Follicular maturation/GC activity	CD21/CD23; BCL6; Ki-67	Detection of follicular dendritic cell networks and proliferative GC reactions [1,17,18,57,70].	Supports mature TLS and structural TLS-A candidacy; incomplete or absent GC features support TLS-B.
High endothelial venules	PNAd/MECA-79	Assessment of lymphocyte recruitment infrastructure and lymphoid organisation [1,18,43,58,70].	Supports TLS organisation and maturation, but does not define functional state alone.
Effector immune context	CD8; granzyme B; DC-LAMP/CD208; CD138 ± IgG/IgA	Assessment of cytotoxic activity, mature dendritic cell niches, and plasma cell responses [5,12,19,35,38,42,60,62].	Supports TLS-A when spatially linked to GC-positive TLS and tumour-facing immune activation.
Regulatory lymphoid context	FOXP3/CD25; Breg-like markers where available; IL-10/PD-L1-related features	Identification of Treg- or Breg-associated suppressive programs [5,12,30,31,32].	Supports TLS-C only when regulatory cells/programs are enriched within or around the TLS niche.
Suppressive myeloid context	CD68, CD163, CD206; MPO or CD66b	Assessment of macrophage-rich, M2-like, neutrophil-rich, or MDSC-like suppressive niches [36,37,38,39,40,44,48].	Supports TLS-C when spatially linked to TLSs and accompanied by impaired effector activity or other suppressive features.
Checkpoint/metabolic suppression	PD-L1, CTLA-4, LAG-3, TIM-3, IDO1	Detection of checkpoint-rich or metabolically suppressive immune context [5,31,32,43,44,58,64,65].	Supports TLS-C when localised to TLSs or TLS borders and interpreted together with cellular context.
Optional molecular support	TLS/GC/Tfh/B-cell, plasma cell, IFN/cytotoxic, suppressive myeloid, checkpoint, or T-cell dysfunction signatures	Molecular support for structural and immune context interpretation [5,9,43,58,64,65,103].	Supports but should not replace morphology, spatial localisation, and immune context assessment.

**Table 4:** Tiered implementation of the proposed Functional TLS Score according to available pathology and molecular resources.

Assessment Level	Practical Assessment	Key Variables/Biomarkers	Possible Output	Main Limitation
Low-resource/basic pathology	H&E/HES + CD20/CD19 and CD3.	TLS presence; loose aggregate vs. organised TLS; basic B-/T-cell organisation; approximate localisation.	TLS present/absent; structural maturity estimate; low-confidence TLS-B or TLS-A candidate.	Insufficient for confident TLS-C assignment.
Intermediate pathology	Expanded IHC panel.	CD21/CD23, BCL6, Ki-67, PNAd/MECA-79, CD8, CD138, FOXP3, CD68/CD163 ± CD206, MPO/CD66b, selected checkpoint markers [1,5,17,18,31,32,43,44,56,57,58].	Preliminary TLS-A/B/C assignment when maturity, localisation, and immune context are concordant.	Limited spatial resolution; no validated thresholds.
Advanced translational setting	Multiplex IHC/IF, digital pathology, spatial proteomics/transcriptomics, RNA-based TLS signatures.	TLS–tumour distance; immune neighbourhoods; effector/suppressive programs; TLS/GC/Tfh/B-cell, IFN/cytotoxic, checkpoint, myeloid, and dysfunction signatures.	High-confidence functional assignment; detection of mixed or region-specific TLS states.	Requires specialised infrastructure and prospective validation.
Final reporting	Integrated interpretation of structural maturity, burden, localisation, immune context, and optional molecular support.	Predominant TLS state; secondary/mixed states; confidence level; unclassifiable cases.	TLS-A, TLS-B, TLS-C, mixed TLS state specimen, or unclassifiable TLS.	Conceptual framework; not a validated clinical score.

**Table 5:** Minimum reporting checklist for standardised assessment and functional assignment of tertiary lymphoid structures in solid tumours.

Reporting Item	What to Report	Why It Matters
Specimen context	Biopsy/resection; pre- or post-treatment	TLS may change with sampling and therapy
TLS definition	GC-positive mTLS, non-GC TLS, loose lymphoid aggregate	Avoids mixing immature and mature TLSs
Staining method	H&E/HES, IHC, multiplex, spatial, RNA	Defines the level of evidence
Marker panel	B/T cell, FDC, GC, HEV, effector/suppressive markers	Supports structural and functional assignment
TLS burden	Count, density, area, or maturity-weighted score	Quantifies TLSs, but does not define function alone
Location	Intratumoural, invasive margin, peritumoural	Modifies interpretation of TLS function
Immune context	Effector, mixed, or suppressive profile	Distinguishes TLS-A, TLS-B, and TLS-C
Molecular support	TLS, GC/Tfh, IFN/cytotoxic or suppressive signatures	Provides optional supportive evidence
Final category	TLS-A, TLS-B, TLS-C, mixed TLS state specimen, or unclassifiable TLS	Summarises functional assignment

## Data Availability

No new data were created or analyzed in this study. Data sharing is not applicable to this article.

## Abbreviations

The following abbreviations are used in this manuscript:

APC	Antigen-presenting cell
BCL6	B-cell lymphoma 6
BCR	B-cell receptor
Breg	Regulatory B cell
CAF	Cancer-associated fibroblast
CCL	C-C motif chemokine ligand
CCR7	C-C chemokine receptor 7
cDC1	Conventional type 1 dendritic cell
CD	Cluster of differentiation
CD40L	CD40 ligand
CRC	Colorectal cancer
CSR	Class-switch recombination
CSF-1	Colony-stimulating factor 1
CTC	Circulating tumour cell
CTLA-4	Cytotoxic T-lymphocyte-associated protein 4
CXCL	C-X-C motif chemokine ligand
CXCR	C-X-C motif chemokine receptor
DC	Dendritic cell
DC-LAMP	Dendritic cell lysosome-associated membrane glycoprotein
E-TLS	Early tertiary lymphoid structure
FasL	Fas ligand
Fcγ	Fragment crystallisable gamma
FDC	Follicular dendritic cell
FOXP3	Forkhead box P3
FRC	Fibroblastic reticular cell
GC	Germinal centre
H&E	Haematoxylin and eosin
HCC	Hepatocellular carcinoma
HEV	High endothelial venule
HES	Haematoxylin, eosin, and saffron
HGSC	High-grade serous ovarian carcinoma
HIF	Hypoxia-inducible factor
HLA	Human leukocyte antigen
ICB	Immune checkpoint blockade
ICI	Immune checkpoint inhibitor
ICP	Immune checkpoint protein
ICOS	Inducible T-cell co-stimulator
IDO	Indoleamine 2,3-dioxygenase
IDO1	Indoleamine 2,3-dioxygenase 1
IF	Immunofluorescence
IFN	Interferon
IFN-γ	Interferon gamma
IgA	Immunoglobulin A
IgE	Immunoglobulin E
IgG	Immunoglobulin G
IgM	Immunoglobulin M
IHC	Immunohistochemistry
IL	Interleukin
LIGHT	TNF superfamily member 14
LTα	Lymphotoxin alpha
LTβ	Lymphotoxin beta
LTα1β2	Lymphotoxin α1β2 heterotrimer
LTβR	Lymphotoxin beta receptor
LTi	Lymphoid tissue inducer cell
LTo	Lymphoid tissue organiser cell
mDC	Myeloid dendritic cell
MDSC	Myeloid-derived suppressor cell
MHC	Major histocompatibility complex
MMP	Matrix metalloproteinase
MPO	Myeloperoxidase
mTLS	Mature tertiary lymphoid structure
NET	Neutrophil extracellular trap
NK	Natural killer
NSCLC	Non-small-cell lung cancer
oHSV	Oncolytic herpes simplex virus
OS	Overall survival
PC	Plasma cell
PD-1	Programmed cell death protein 1
PDAC	Pancreatic ductal adenocarcinoma
PD-L1	Programmed death ligand 1
PFL-TLS	Primary follicle-like tertiary lymphoid structure
pIgR	Polymeric immunoglobulin receptor
PFS	Progression-free survival
PNAd	Peripheral node addressin
RCC	Renal cell carcinoma
RNA	Ribonucleic acid
SFL-TLS	Secondary follicle-like tertiary lymphoid structure
SHM	Somatic hypermutation
SLO	Secondary lymphoid organ
TA-TLS	Tumour-associated tertiary lymphoid structure
TAM	Tumour-associated macrophage
TAN	Tumour-associated neutrophil
TCR	T-cell receptor
Tfh	T follicular helper cell
TGF-β	Transforming growth factor beta
Th	T helper cell
TIB	Tumour-infiltrating B cell
TIM-1	T-cell immunoglobulin and mucin domain 1
TIM-3	T-cell immunoglobulin and mucin-domain containing 3
TLR	Toll-like receptor
TLS	Tertiary lymphoid structure
TLS-A	Pro-immunogenic effector tertiary lymphoid structure
TLS-B	Transitional/intermediate tertiary lymphoid structure
TLS-C	Immunoregulatory/suppressive tertiary lymphoid structure
TME	Tumour microenvironment
TNF	Tumour necrosis factor
TNFR1	Tumour necrosis factor receptor 1
Tph	T peripheral helper cell
TRAIL	TNF-related apoptosis-inducing ligand
Treg	Regulatory T cell
VCAM-1	Vascular cell adhesion molecule 1
VEGF-C	Vascular endothelial growth factor C
VISTA	V-domain immunoglobulin suppressor of T-cell activation
VISTA-PSGL-1	VISTA–P-selectin glycoprotein ligand 1 pathway
